# Ginseng-derived nanoparticles reprogram macrophages to regulate arginase-1 release for ameliorating T cell exhaustion in tumor microenvironment

**DOI:** 10.1186/s13046-023-02888-7

**Published:** 2023-11-28

**Authors:** Yan Lv, Mengyuan Li, Ling Weng, Haoying Huang, Yujie Mao, Danchen Aaron Yang, Qingyun Wei, Mengmeng Zhao, Qin Wei, Ke Rui, Xuan Han, Weiwei Fan, Xueting Cai, Peng Cao, Meng Cao

**Affiliations:** 1 Jiangsu Provincial Medical Innovation Center, Affiliated Hospital of Integrated Traditional Chinese and Western Medicine, Nanjing University of Chinese Medicine, Nanjing, Jiangsu China; 2 Department of Pharmacology, School of Pharmacy, Nanjing University of Chinese Medicine, Nanjing, Jiangsu China; 3 College of Veterinary Medicine, Nanjing Agricultural University, Nanjing, Jiangsu China; 4 Institute of Medical Immunology, Affiliated Hospital of Jiangsu University, Zhenjiang, Jiangsu China; 5 The Third Affiliated Hospital of Guangzhou University of Chinese Medicine, Guangzhou, Guangdong China; 6 Zhenjiang Hospital of Chinese Traditional and Western Medicine, Zhenjiang, Jiangsu, China

**Keywords:** Ginseng-derived nanoparticles, Arginase-1, Tumor-associated macrophages, T cell exhaustion, mTOR

## Abstract

**Background:**

Lines of evidence indicated that, immune checkpoints (ICs) inhibitors enhanced T cell immune response to exert anti-tumor effects. However, T cell exhaustion has been so far a major obstacle to antitumor immunotherapy in colorectal cancer patients. Our previous studies showed that ginseng-derived nanoparticles (GDNPs) inhibited the growth of various tumors by reprograming tumor-associated macrophages (TAMs) and downregulated the ICs expression on T cells in tumor microenvironment (TME), but the underlying effector mechanisms remained unclear.

**Methods:**

The correlation between arginase-1 (ARG1) and T cells was computed based on the colorectal cancer patients in TCGA database. In vitro, we observed that GDNPs reprogrammed TAMs inhibited ARG1 release and ultimately ameliorated T cell exhaustion according to several techniques including WB, PCR, ELISA and flow cytometry. We also used an in vivo MC38 tumor-bearing model and administered GDNPs to assess their anti-tumor effects through multiple indices. The mechanism that GDNPs improved T cell exhaustion was further clarified using the bioinformatics tools and flow cytometry.

**Results:**

GDNPs reprogramed TAMs via reducing ARG1 production. Moreover, normalized arginine metabolism ameliorated T cell exhaustion through mTOR-T-bet axis, resulting in reduced ICs expression and enhanced CD8^+^ T cells expansion.

**Conclusions:**

By regulating the mTOR-T-bet axis, GDNPs reprogramed macrophages to regulate ARG1 release, which further ameliorated T cell exhaustion in TME. These findings provided new insights into comprehending the mechanisms underlying the mitigation of T cell exhaustion, which may facilitate the development of innovative therapeutic strategies in the field of cancer treatment.

**Supplementary Information:**

The online version contains supplementary material available at 10.1186/s13046-023-02888-7.

## Background

Recent advances in tumor immunotherapy, specifically targeting PD-1, CTLA4, and other immune checkpoints, have shaped the paradigm shift in cancer treatment with durable clinical responses [1]. Nevertheless, the benefits from the immune checkpoint inhibitor therapy were not evident in colorectal cancer patients [2, 3]. Subsequently, the tumor microenvironment (TME) of many patients continued to deteriorate with the ongoing expression of immune checkpoints on the surface of T cells, ultimately resulting in T cell exhaustion [4]. Many studies have attempt to elucidate the mechanisms underlying T cell exhaustion in the field of colorectal cancer treatment.

Arginase1 (ARG1), a representative marker of M2-like macrophages [5], has been shown to inhibit T cell immune response and accelerate tumor growth [6]. It was reported that ARG1 was involved in the arginine metabolic pathway, where arginine was broken down into urea and ornithine whereas proline and polyamines were generated downstream, ultimately resulting in T cell dysfunction [7]. The main source of ARG1 is myeloid cells, among which tumor-associated macrophages (TAMs) are an important source of ARG1 [8]. Current research suggested that ARG1 was over-expressed in tumors at the stage of rapid tumor development [9, 10], which strongly impeded the function of T cells and presents a state of exhaustion, but its relevance to immune checkpoint has not been thoroughly studied.

Our previous studies reported that the extracellular vesicle-liked nanoparticles extracted from fresh ginseng (Ginseng-derived nanoparticles, GDNPs) could reprogram TAMs to reduce the production of ARG1 [11]. Furthermore, our research revealed that GDNPs treatment decreased the expression of immune checkpoints (PD-1, TIM3 and ICOS), while simultaneously promoting the recruitment of more activated T cells to the TME [12]. Building upon these earlier findings we hypothesized that the reduction in ARG1 production as a result of GDNPs treatment could have further influence on the expression of immune checkpoints. Therefore, further investigation was required to understand whether GDNPs could lower the ARG1 expression in order to ameliorate T cell exhaustion. In this study, we discovered a close association between ARG1 and T cell exhaustion. We also elucidated the mechanism through which GDNPs reprogrammed TAMs to diminish ARG1 production, thereby improving T cell exhaustion within the TME. The reduction of ARG1 production maintained normal L-Arginine level in TME, which subsequently activated mTOR-T-bet pathway. This activation, in turn, led to the downregulation of immune checkpoint expressions. T cell exhaustion was improved with enhanced cytotoxic activity by GDNPs, indicating the potential application of GDNPs for the treatment of colorectal cancer.

## Materials and methods

The GDNPs preparation and characterization, RNA isolations, quantitative real-time (q) PCR, western blotting, single-cell sequencing assay, spatial metabolomics assay and assay of arginine on T cells are presented in online supplemental materials and methods.

### Mice and cell lines

Six to eight-weeks old male C57BL/6 mice were purchased from the Comparative Medicine Center, Yangzhou University (Yangzhou, Jiangsu, China). All animal experimental protocols were approved by the Institutional Animal Care and Use Committee of Affiliated Hospital of Integrated Traditional Chinese and Western Medicine, Nanjing University of Chinese Medicine (AEWC-20210528–148).

The murine colon cancer cell line MC38 was purchased from the Institute of Biochemistry and Cell Biology, Academy of Science (Shanghai, China). Cells were cultured in dulbecco’s modified eagle medium (DMEM), RPMI 1640 or RPMI 1640 without L-Arginine, supplemented with 10% fetal bovine serum (FBS), 100 U/mL penicillin, and 100 mg/mL streptomycin (all from Thermo Fisher Scientific, USA). All cells were incubated at 37 °C in a humidified atmosphere with 5% CO_2_.

### Study design

This study was designed to characterize the effect of GDNPs by analyzing samples collected from the mice with tumor. Mouse MC38 tumor model was selected to evaluate the treatment efficiency. Six to eight-weeks old male mice were inoculated subcutaneously with 5 × 10^5^ cell in the right upper flank (*n* = 5 per group, Day 0). The first treatment was scheduled until the tumor was around 50–100 mm^3^ on day 7. The vehicle group received PBS 100 μL per mouse. GDNPs (250 μg per mouse) were injected intraperitoneally (i.p.) on day 7, 10, 13, 16 and 19 in the GDNPs group. The tumors were measured every other day with a caliper and the volume was calculated (length × width^2^/2). Mice were euthanized in a CO_2_ chamber and sacrificed when tumor volume was over 1500 mm^3^. Tumor weight was calculated using an electronic weighing machine.

### Mouse bone marrow-derived macrophages (BMDM) preparation and polarization assay

Bone marrow derived monocytes were isolated from tibia and femurs of male C57BL/6 mice (6–8 weeks old). To polarize monocytes to macrophages, cells were cultured in DMEM medium with 10% FBS and 20 ng/mL M-CSF for every 3 days after red cell lysis. 20 ng/mL IL-4 and 20 ng/mL IL-13 were added to polarize M0 to M2-like macrophages for 48 h. Twenty ng/mL IFN-γ and 50 ng/mL LPS were added to polarize M0 to M1-like macrophages for 48 h. Cells were prepared for the qPCR, western blot and enzyme-linked immunosorbent assay (ELISA).

### ELISA assay and arginine level assay

After 48 h, collected supernatant of M0, M1, M2 and M2 + GDNPs groups to detect ARG1 concentration by ELISA in accordance with the manufacturer’s instructions.

M2-like macrophages stimulated or not stimulated by GDNPs were prepared by the previous method, and supernatants of M2 and M2 + GDNPs were collected for 48 h in vitro. Then, tumors in the vehicle and GDNPs groups were ground and tumor supernatant was collected. These samples were detected by L-Arginine Assay Kit (ab241028), following the manufacturer's instructions.

### T cell activation and proliferation assay

M2-like macrophages (5 × 10^5^/well) were incubated with or without GDNPs (10 μg/mL), after 48 h, add nor-NOHA (10 ng/mL) or L-Arginine (1 mM), the supernatant after 48 h was collected and cultivated the splenocytes. The spleens were scissored into 5 mm pieces and passed through a 40 μm filter. Following the removal of red blood cells through lysis (C3702, Beyotime), the remaining cells were resuspended in RPIM 1640 medium for future use.

IFN-γ concentration in the supernatant was quantified by ELISA in accordance with the manufacturer’s instructions. Besides, splenocytes were gathered for flow cytometry to detect exhaustion indicators on the surface of T cells, transcription factors in T cells.

The culture system was the same as the ELISA assay for IFN-γ concentration. Splenocytes were labeled with Cell Trace CFSE dye (2 μM final concentration, Thermo Fisher Scientific) according to manufacturer’s manual. The labeled splenocytes were plated in round-bottomed 24-well plates (1–2 × 10^6^/well) in the supernatant of M2, M2 + GDNPs, M2 + nor-NOHA and M2 + L-Arg groups stimulated with anti-CD3/CD28 (0.5 μg/mL) (Table S2). After incubation for 72 h at 37 °C, 5% CO_2_ splenocytes were harvested, stained with anti-CD3-APC/Cy7, anti-CD4-APC, or anti-CD8-BV421 (Table S1) and analyzed by FACS Aria II system (BD Biosciences, USA).

### Isolation of tumor-infiltrating cells and splenocytes

Mouse tumor tissues were scissored into 5 mm pieces and incubation with 100 μg/mL Liberase and 0.2 mg/mL DNase I in PBS for 45 min at 37 °C. After digestion, 70 μm nylon net was centrifuged at 300 g for 5 min. Samples are used for flow cytometry analysis.

Splenocytes were prepared by methods similar to those previously described [10, 13]. The spleens finely minced into 5 mm pieces and then filtered through a 40 μm mesh. After red blood cell lysis (C3702, Beyotime), the cells were resuspended in RPIM 1640 medium for future analysis.

### Flow cytometry of immune cells in the TME and splenocytes

Immune cells were isolated from tumors using percoll (17–0891-09; GE Healthcare). The cells were incubated with CD16/32 (clone: 93, Biolegend) for 20 min on ice and then were stained with various combinations of following fluorochrome-conjugated antibody at the appropriate dilutions for 30 min on ice, namely, FVD506, CD3-APC/Cy7, CD3-APC, CD8a-PE, CD8a-BV421, CD45-FITC, CD11b-APC/Cy7, F4/80-BV421, CD86-PE, CD206-APC, CD4-APC, CD4-PE/Cy7, CD11c-PE, NK1.1-Percp-Cy5.5, TIM3-PE, ICOS-PE/Cy7, PD-1-Percy 710, TIGIT-BV421 (Table S1).

After labeling markers on the cell surface, the Fixation/Permiablization kit (00–5123-43, 00–5223-56; Invitrogen) were used as manufacture’s instruction. Subsequently, ARG1-APC, Ki67-Percp-Cy5.5, Tox-PE, T-bet-PE/Cy7, Eomes-PE (Table S1) were diluent in Permeabilization buffer 10 × (1:10) for 45 min.

Another protocol was used for intracellular staining of phosphorylated proteins. After 30 min of staining with live or dead dye, surface markers: CD3-APC/Cy7, CD4-APC, CD8-BV421 (Table S1) were stained for 30 min. Then, the cells were immobilized and broken for 45 min, and stained p-S6, p-4EBP1 for 60 min. Finally, the second antibody (Anti-rabbit IgG (H + L), F(ab')2 Fragment (Alexa Fluor® 488 Conjugate)) were stained for 60 min.

Stained cells were analyzed on a FACS Aria II Flow Cytometer, BD Biosciences) using BD FACS Diva software (BD Bioscience, USA) and data were processed using Flowjo Version 10 (BD Bioscience, USA).

### Inhibition of mTOR activity assay

Splenocytes were cultured with the supernatant of M2 macrophages stimulated with or without GDNPs and rapamycin treated splenocytes were cultured with the supernatant of M2 macrophages stimulated with GDNPs for 48 h.

The cells were collected for flow cytometry to detect mTOR activity (p-S6, p-4EBP1), exhaustion indicators and transcription factors.

### T cell transfusion assay

Mouse spleens were prepared into cell suspensions by the method mentioned above. The CD8^+^ T cells were selected from splenocytes, using the CD8^+^ T cell Dissociation Kit (130–117-044, MACS) as per the manufacturer’s instructions. CD8^+^ T cells treated with or without rapamycin were cultured from the supernatant of M2-like macrophages stimulated or not by GDNPs or of M2-like macrophages with or without arginine for. Then, these T cells (2 × 10^6^ /mouse) were transfused into MC38-bearing mice model in intravenous injections (i.v.)on the day 7. After the mice were sacrificed on the 21st day, single-cell suspensions of the tumor tissue were prepared. The cells were collected for flow cytometry to detect mTOR activity (p-S6, p-4EBP1), transcription factors (T-bet). The detailed flow cytometry detection methods can be found by referring to the previous description.

### Bioinformatic analyses

Use the online platform TIMER 2.0 (http://timer.cistrome.org/) to analyze ARG1 expression and analyze the correlation between ARG1 and immune checkpoint ICOS, PD-1, TIGIT and TIM3 in tumors.

### Statistical analysis

The results were expressed as the mean ± standard error (S.E.M). All data were analyzed using GraphPad Prism 8.0 (GraphPad Software, USA) by unpaired Student’s test, one-way and two-way analysis of variance (ANOVA). *P* < 0.05 was considered statistically significant (^*^P < 0.05, ^**^P < 0.01, ^***^P < 0.001, ^****^P < 0.0001).

## Results

### ARG1 was closely associated with tumor progression and increased immune checkpoints in T cells

**Fig. 1:**
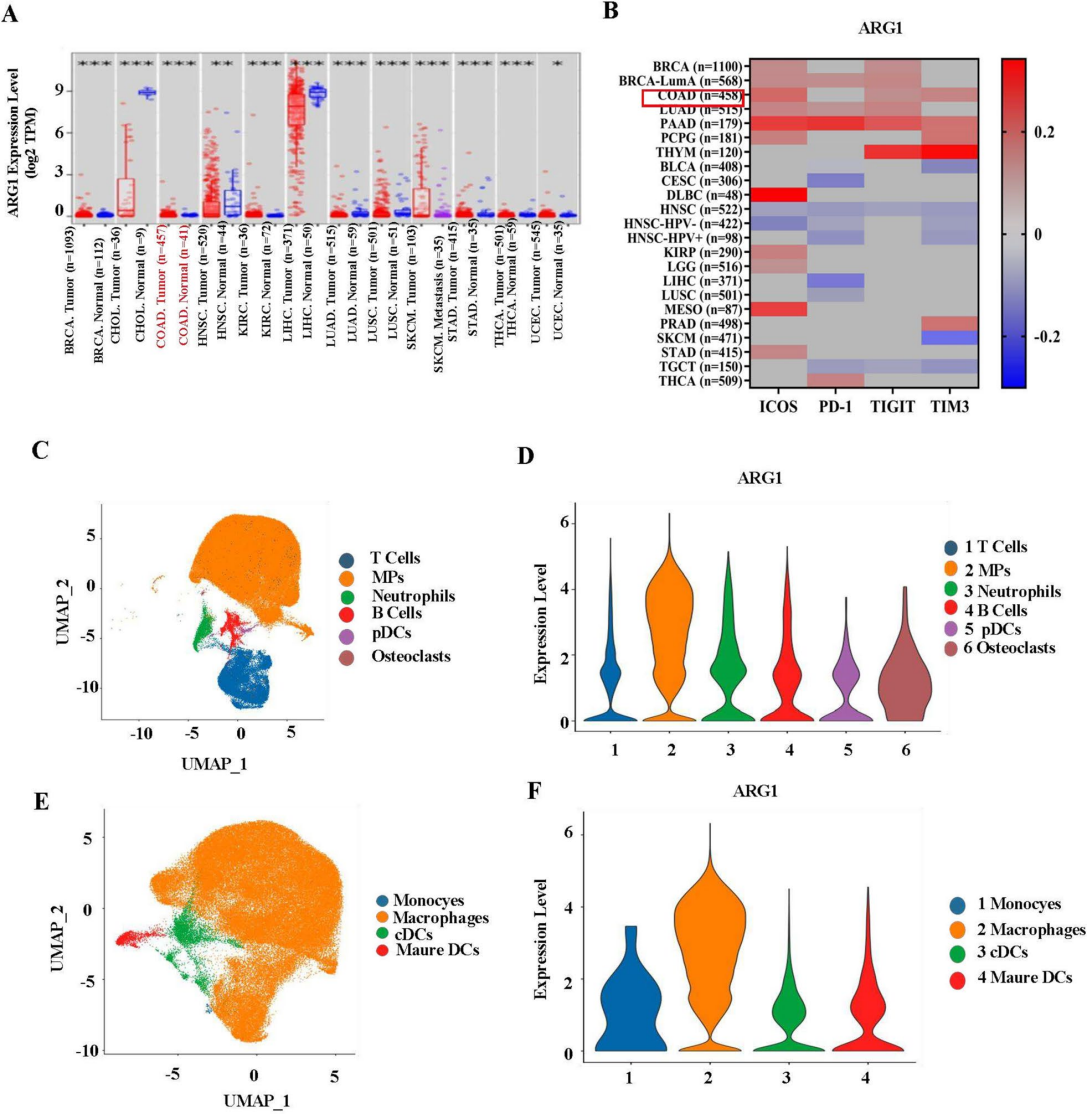
ARG1 was closely associated with tumor progression and increased immune checkpoints expression on T cells. **a** ARG1 expression between tumor and normal samples analyzed by TIMER 2.0 (http://timer.cistrome.org/). **b** The correlation between ARG1 and immune checkpoint ICOS, PD-1, TIGIT and TIM3 in various tumors was analyzed by heat map (*p* < 0.05). Download TCGA data on TIMER 2.0 (http://timer.cistrome.org/) platform. **c** Six major immune cell types in MC38 tumors were identified by UMAP analysis. **d** Feature plots for expression of Arg1 in six kinds of immune cells. **e** Four main cell types in MPs were identified by UMAP analysis. **f** Dot plots showing the Arg1 gene expression in four kinds of MPs

In TME, high levels of ARG1 expression promoted tumor growth and metastasis [11]. We employed the online platform TIMER 2.0 (http://timer.cistrome.org/) to analyze ARG1 expression in various tumors. The results showed that high expression of ARG1 was detected in colorectal cancer, breast cancer, skin melanoma and other tumors (Fig. 1A). We also used the GSE4107 dataset to analyze the expression level of ARG1 in colorectal cancer, which was consistent with the current results, and ARG1 was highly expressed in colorectal cancer (Supplementary Fig. 1A). In addition, ARG1 mediated tumor escape to promote rapid tumor growth [14]. Higher ARG1 expression was associated with lower survival probability, suggesting a link to poor prognosis in colorectal cancer [15]. Moreover, ARG1 inhibited T cell immune response [6, 9]. This was also supported by our findings that at least three positive correlations between ARG1 and immune checkpoints in colon, breast, lung, and pancreatic cancers were observed (Fig. 1B). In summary, ARG1 was positively correlated with TIM3, TIGIT and ICOS in colorectal cancer.

In addition, the immunofluorescence (IF) staining revealed that ARG1 were mainly expressed in F4/80^+^ macrophages (Supplementary Fig. 1B) indicating that TAMs might be an important source of ARG1 production in MC38 tumors. Furthermore, flow cytometric analysis showed that ARG1 was mainly expressed in CD45^+^ cells in MC38 tumor (Supplementary Fig. 1C), suggesting that the majority of ARG1 was derived from CD45^+^ cells. Single cell sequencing analysis of immune cells in TME showed that ARG1 was mainly expressed in mononuclear phagocytics system (MPs) cells, but hardly expressed in T cells, dendritic cells (DC) and natural killer cells (NK) (Fig. 1C, D) (Supplementary Fig. 1F). Further analysis found that MPs cells contained monocytes, macrophages, conventional type 2 dendritic cells (cDCs) and mature DCs with macrophages being the predominant component atan average ratio of 42.12 (Fig. 1E) (Supplementary Fig. 1D, E). The analysis of ARG1 expression of these four types of cells illustrated that the production of ARG1 was mainly from macrophages (Fig. 1F). Together, these data indicated that ARG1, primarily derived from TAMs, was associated with immune checkpoints and T cell exhaustion in colorectal cancer.

### GDNPs reprogrammed TAMs to reduce ARG1 production

**Fig. 2:**
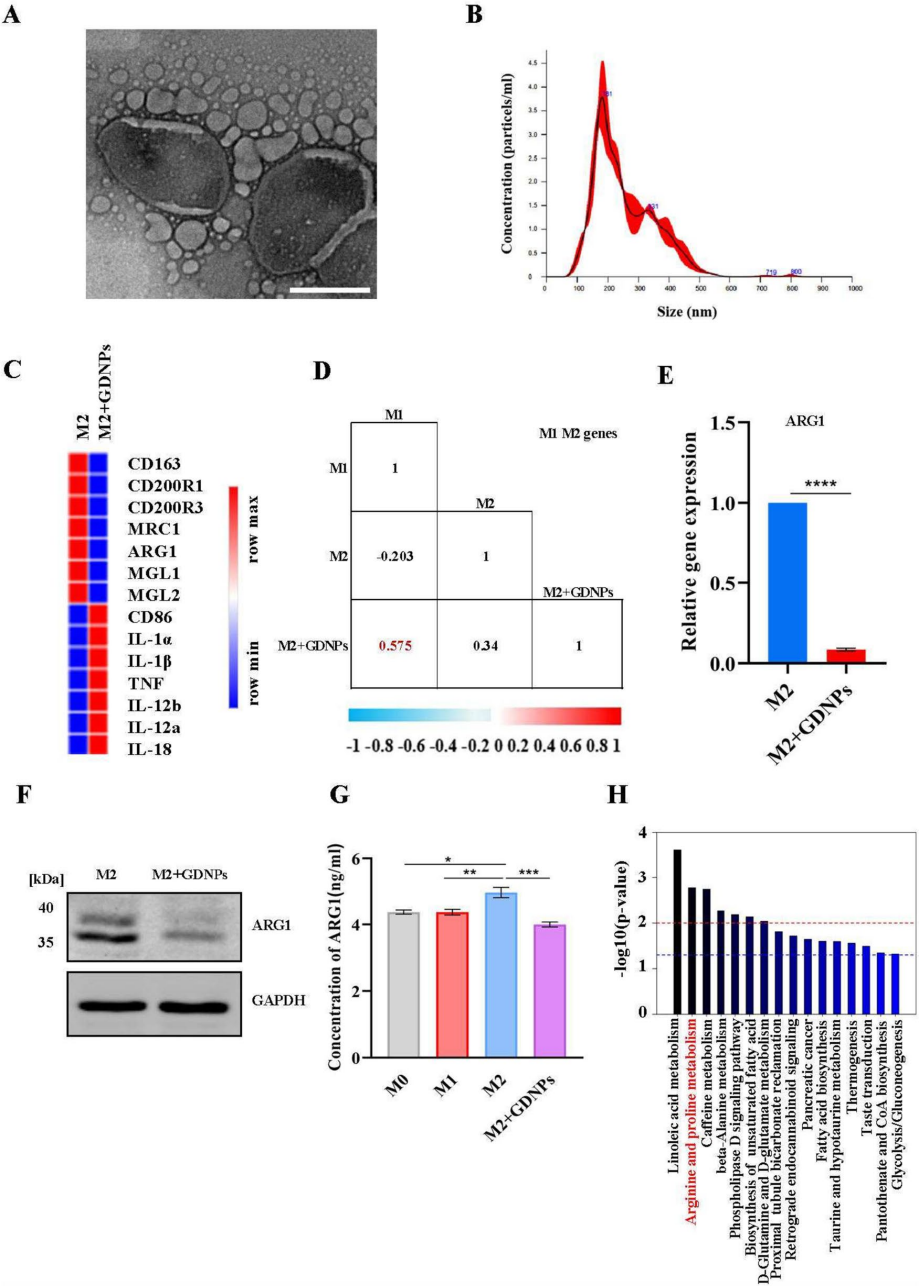
GDNPs reprogrammed TAMs to reduce ARG1 production. **a** TEM image of GDNPs were purified with differential ultracentrifugation. **b** The size and concentration of GDNPs were detected by NTA technology. **c** Heat map analysis of M1-M2 related genes expression from M2-like macrophages treated with or without GDNPs. **d** Pearson’s correlation analysis of gene expression matrices between samples using M1-M2 markers set derived from M2-like macrophages. Values range from 0 to 1, where a high value indicates high degree of correlation between two sample sets. **e** q-PCR analysis of Arg1 mRNA expression in M2 macrophages treated with or without GDNPs (10 μg/mL) for 12 h. **f** Expression of ARG1 in the M2 and M2 + GDNPs are detected by western blotting. **g** Concentration of ARG1 in the supernatants was analysed by ELISA. **h** The histogram showed metabolite-enriched KEGG pathways in tumors of wild-type tumor-bearing mice and GDNPs-treated mice. The results represent three independent experiments as the mean ± SEM. One-way ANOVA (g) and Student’s t test (e, f) were used to compare results of different experimental groups for statistically significant difference (*P < 0.05, **P < 0.01, ***P < 0.001)

It was shown that M2-like macrophages accounted for the majority of TAMs [16]. Our previous studies showed that GDNPs effectively inhibited the growth of several tumors by reprogramming TAMs to M1-like macrophages in mice [11, 12]. To further examine the effect of GDNPs on macrophages, GDNPs were prepared and examined by transmission electron microscopy (TEM), a double-layer structure of GDNPs was observed (Fig. 2A) with an average particle size = 254.6 nm by nanosight tracking analysis (NTA) (Fig. 2B). Next, the whole-transcriptome RNA sequencing (RNA-seq) was performed on M2-like macrophages treated with or without GDNPs (Table S6). The Results demonstrated that the presence of GDNPs led to upregulation of M1-related genes and downregulation of M2-related genes in M2-like macrophages (Fig. 2C). Subsequently, Pearson's correlation analysis showed that M2-like macrophages treated by GDNPs were similar to M1-like macrophages, indicating that GDNPs reprogrammed M2-like macrophages into M1-like macrophages (Fig. 2D). In addition, particular attention was directed towards the expression of ARG1, a representative marker of M2-like macrophages. Real-time quantitative PCR assay (Fig. 2E) and western blotting assay (Fig. 2F) (Supplementary Fig. 2A) indicated a reduced production of ARG1 in M2-like macrophages when treated with GDNPs. ELISA results, which measured ARG1 concentration in the supernatant of different phenotypes, showed that GDNPs inhibited M2-like macrophages releasing ARG1 (Fig. 2G). Spatial metabolomics data indicated significant alterations in arginine metabolic pathways within the colon tumor model due to GDNPs (Fig. 2H), potentially linked to the reduction of ARG1 releasing during GDNPs-induced macrophage reprogramming. Overall, our findings highlighted that GDNPs downregulated ARG1 production by polarizing M2-like to M1-like macrophages.

### GDNPs reduced ARG1 production to promote proliferation and activation of T cells by polarizing TAMs.

**Fig. 3:**
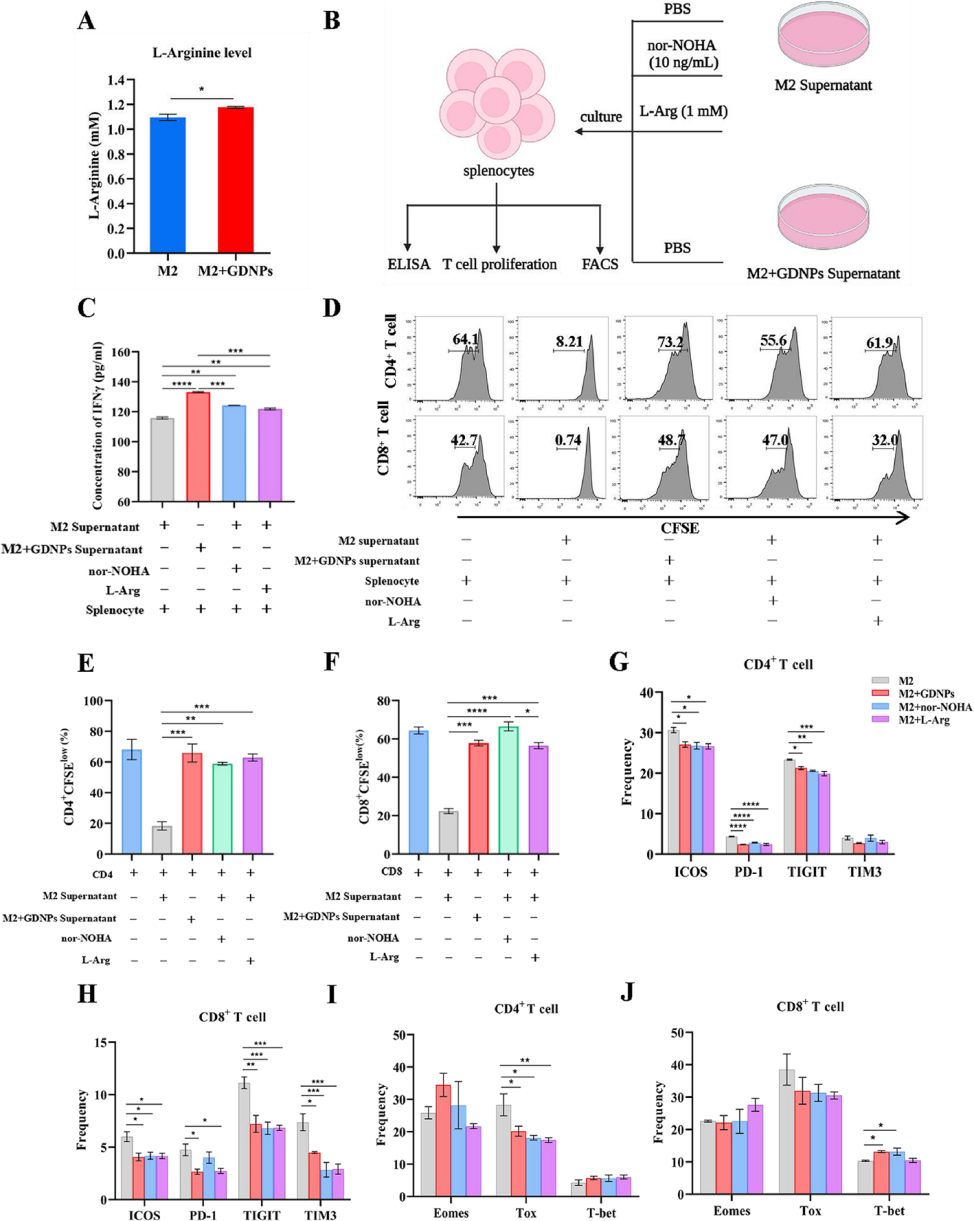
GDNPs reduced ARG1 production to promote proliferation and activation of T cells by polarizing TAMs. **a** L-Arginine level in supernatants from M2 and M2 + GDNPs was detected by L-Arginine Assay Kit. **b** Schematic diagram of culture system. **c** IFN-γ concentration in the supernatants was analysed by ELISA. **d** ARG1 inhibited the proliferation of CD4 + and CD8 + T cells. Splenocytes were cultured with M2 supernatant supplemented with PBS or nor-NOHA (10 ng/mL) or L-Arginine (1 mM) and M2 + GDNPs supernatant for 72 h. Representative histograms of CD4 + and CD8 + T cells proliferation. **e**, **f** Quantification of CD4 + and CD8 + T cells proliferation using CFSE dilution. **g**, **h**, **i**, **j** Splenocytes were cultured with M2 supernatant supplemented with PBS or nor-NOHA (10 ng/mL) or L-Arginine (1 mM) and M2 + GDNPs supernatant for 48 h. **g**, **h** Representative quantification of immune checkpoints of ICOS, PD-1, TIGIT, and TIM3 on CD4 + and CD8 + T cells. (**i, j**) The expression of transcription factors Eomes, Tox and T-bet in CD4 + and CD8 + T cells were analyzed. The results represent three independent experiments as the mean ± SEM. One-way ANOVA (**c**, **g**, **h**, **i**, **j**) and Student’s t test (**a**, **e**, **f**) were used to compare results of different experimental groups for statistically significant difference (*P < 0.05, **P < 0.01, ***P < 0.001, ****P < 0.0001)

ARG1 is involved in the arginine metabolic pathway and decomposes arginine [17]. Arginine metabolism is important for the activity and response of immune cells, especially T cells [18]. Here, we found that the level of arginine in the supernatant of M2-like macrophages was higher following treatment with GDNPs compared to the untreated group (Fig. 3A). We established a culture system to explore whether GDNPs could sustain the arginine level in TME and enhance T cells immune response. This was achieved by culturing splenocytes with the supernatant of M2-like macrophages, with or without GDNPs treatment, along with nor-NOHA or L-Arginine (1 mM) (Fig. 3B). The highest IFN-γ secreted by splenocytes was found in GDNPs treatment group (Fig. 3C). As shown by T cell proliferation assay, the suppression of CD4^+^ and CD8^+^ T cells was mitigated when GDNPs were added to the M2-like macrophages (Fig. 3D), and the proliferation rate was 3 times higher than that of the untreated M2-like macrophages supernatant (Fig. 3E, F).

Next, we measured the expression of immune checkpoints on T cells incubated with different supernatants of M2-like macrophages using flow cytometry. Our flow cytometry results revealed that the presence of GDNPs led to a significant decrease in the expression of ICOS, PD-1, TIGIT on both CD4^+^ and CD8^+^ T cells. Additionally, the expression of TIM3 decreased significantly but only on CD8^+^ T cells (Fig. 3G, H). No significant differences in expressions of immune checkpoints on CD4^+^ and CD8^+^ T cells were observed amongst the groups with the GDNPs, nor-NOHA or L-Arg. In order to analyze the cause of immune checkpoint changes, we also detected transcription factors Eomes, Tox and T-bet in T cells. The Tox expression in CD4^+^ T cells was lower in GDNPs treatment group compared to the control group (M2-like macrophages without any additional substances), while the expression in GDNPs treatment group was similar to those of nor-NOHA or L-Arginine (Fig. 3I). The differences of Tox expression in CD4^+^ T cells were not found in CD8^+^ T cells. However, the expression of T-bet in CD8^+^ T cells was higher in GDNPs and nor-NOHA treatment groups compared to that of the control group (Fig. 3J). Collectively, these data demonstrated that macrophage polarization by GDNPs could enhance T cells activation and ameliorate T cells exhaustion, primarily by down-regulating ARG1 level to improve arginine metabolism.

### GDNPs improved arginine metabolism in TME to ameliorate T cell exhaustion

**Fig. 4:**
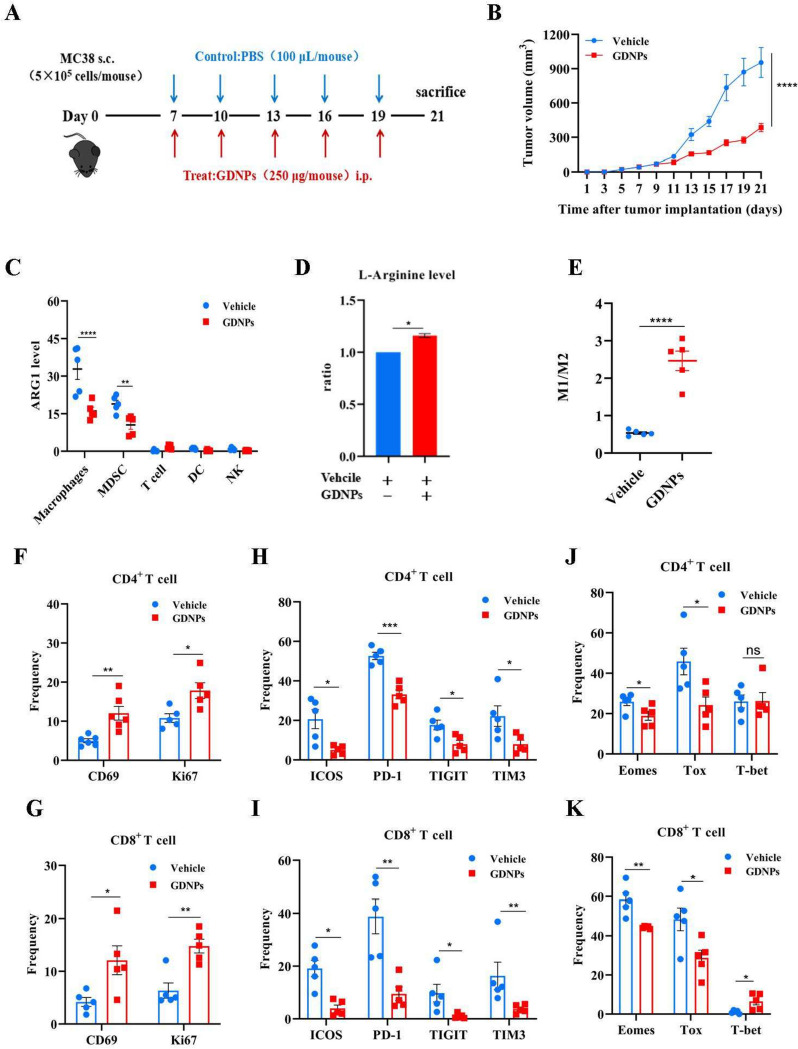
GDNPs improved arginine metabolism in TME to relieve T cell exhaustion. **a** Schematic illustration of GDNPs/Vehicle treatment regimen for MC38 colon tumor model. At 21 days, mice were sacrificed, and the anticancer effects in each group were evaluated and compared (*n* = 5). **b** Tumor volume at the end of the experiment were compared. **c** ARG1 expression in different immune cells in GDNPs/Vehicle groups (*n* = 5). **d** L-Arginine levels in GDNPs/Vehicle treatment of MC38 colon tumor were detected by L-Arginine Assay Kit. **e** The ratio of the proportion of M1-like to M2-like macrophages in the two groups was detected by flow cytometry. **f**, **g** Representative histograms and quantification of CD4 + and CD8 + T cell activation and proliferation in MC38 colon tumors treated with or without GDNPs. **h**, **i** Representative histograms and quantification of surface expression of ICOS, PD-1, TIGIT, and TIM3 on CD4 + and CD8 + T cells in MC38 colon tumors treated with or without GDNPs. **j**, **k** The expression of transcription factors Eomes, Tox and T-bet in CD4 + and CD8 + T cells were analyzed in MC38 colon tumors treated with or without GDNPs. All results represent the mean ± SEM (*n* = 5). Two-way ANOVA (b, c) and Student’s t test (**d**, **e**, **f**, **g**, **h**, **j**, **k**) were used to compare results of different experimental groups for statistically significant difference (*P < 0.05,**P < 0.01,***P < 0.001,****P < 0.0001)

Next, we investigated whether GDNPs treatment had a similar effect in vivo and whether such effect would favor anti-tumor immune response. To this end, we established a tumor-bearing model by subcutaneously inoculating MC38 colon cancer cell into the right flanks of male C57/BL6 mice. Mice were administrated PBS (vehicle) or GDNPs every other day starting on day 7 and the experiment ended on day 21 post-tumor implantation (Fig. 4A). On day 21, the mice were weighed before scarification, and the tumor of each mouse was photographed and weighed. Treatment with GDNPs significantly inhibited tumor growth compared with the vehicle group (Fig. 4B) (Supplementary Fig. 3A, B). However, there was no significant difference in body weight between the two groups (Supplementary Fig. 3D). These results suggested that GDNPs had an anti-tumor effect.

FACS analysis was conducted to assess ARG1 expression in the major immune cells within the TME. The results suggested a significant reduction in ARG1 expression among macrophages and myeloid-derived suppressor cells (MDSC) following treatment with GDNPs (Fig. 4C). Meanwhile, arginine level was higher in the GDNPs group, compared to the vehicle group (Fig. 4D). According to the results of in vitro experiments, this was related to GDNPs reprogramming TAMs and this idea was further supported by the analysis comparing the proportions of TAMs between the GDNPs and vehicle groups. The result showed that the M1/M2 ratio was higher in the GDNPs treatment group (Fig. 4E). These data suggested that GDNPs could inhibit the production of ARG1 by reprogramming TAMs to maintain arginine levels, which promoted its anti-tumor effect.

Generally, exhausted CD8^+^ T cells upregulate an array of co-inhibitory receptors, such as PD-1, ICOS, TIGIT and TIM-3 and establish a unique epigenetic program at gene loci associated with exhaustion, such as Tox [19]. However, little is known about the comprehensive expression programs and functional states of infiltrating T cell arginine metabolism. In order to investigate the interplay among GDNPs, ARG1 and T cell exhaustion, we examined T cell activation, proliferation, and the expression of immune checkpoints. The results from flow cytometry analysis showed that, when compared to the vehicle group, the GDNPs treatment significantly up-regulated the expressions of CD69 and Ki67, while down-regulating the expressions of ICOS, PD-1, TIGIT, TIM-3 on both CD4^+^ and CD8^+^ T cells (Fig. 4F, G, H, I) (Supplementary Fig. 4A). To further determine the potential impact of GDNPs on alleviating T cell exhaustion, we assessed the expressions of transcription factors Eomes, Tox and T-bet in T cells (Fig. 4J, K) (Supplementary Fig. 4B). These data showed that GDNPs suppressed the expressions of Eomes and Tox in both CD4^+^ and CD8^+^ T cells, but it enhanced the expression of T-bet solely in CD8^+^ T cells. In summary, our findings demonstrated that GDNPs exerted an anti-tumor effect by boosting T cell activation, promoting T cell proliferation, and mitigating T cell exhaustion.

### GDNPs ameliorated T cell exhaustion by enhancing the effecting function of CD8 Teff

**Fig. 5:**
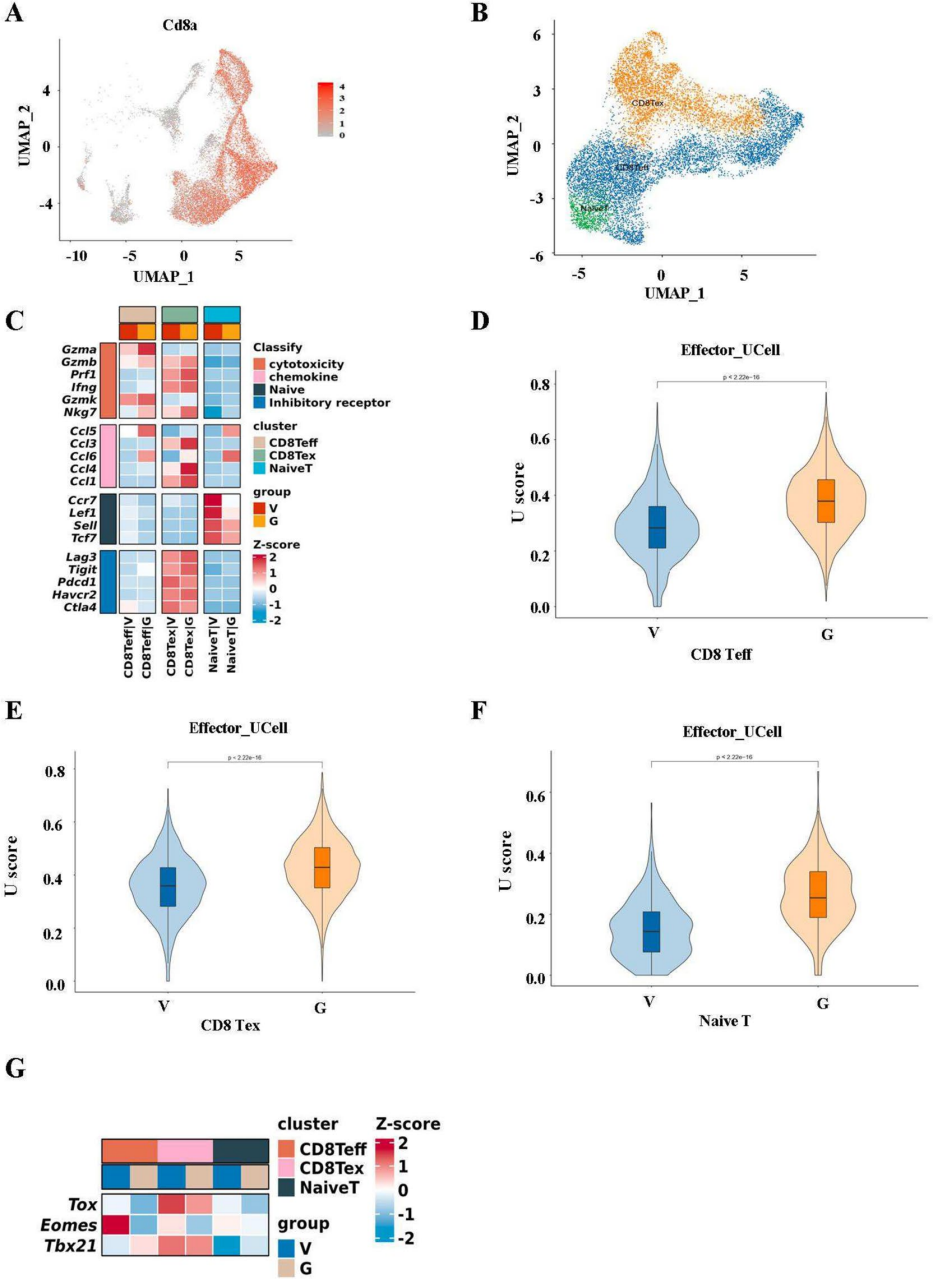
GDNPs relieved T cell exhaustion by enhancing the effecting function of CD8 Teff. **a** UMAP embedding showed expression levels of Cd8a of T cells indicated in panel. **b** UMAP showing the composition of T cells colored by cluster. **c** Heatmap showed the expression of genes in four modules of cvtotoxicity, chemokine, naive and inhibitory receptor in CD8 + T cells subclusters (naive T, CD8 Teff and CD8 Tex) in G (GDNPs) and V (Vehicle) groups. **d**, **e**, **f** Ucell scores were performed for gene sets with effector characteristics in three CD8 + T cells subclusters. **g** Heatmap showed transcription factor (Tox, Eomes, Tbx21) expression in T cell subsets in groups G and V. All results represent the mean ± SEM (*n* = 3). Wilcox (d, e, f) was used to compare results of different experimental groups for statistically significant difference (****P < 0.0001)

To explore how GDNPs influenced CD8^+^ T cell subsets to further mitigate T cell exhaustion, we first used single-cell sequencing to detect changes of T cells with GDNPs administration. Our results showed that GDNPs significantly enhanced T cell number in TME, consistent with the previous results (Supplementary Fig. 5A). We then conducted clustering of CD8^+^ T cells, categorizing them into three distinct subtypes: naive T, CD8 effector T cells (CD8 Teff) and CD8 exhaustion T cells (CD8 Tex) (Fig. 5A, B). The expressions of genes in four modules (cytotoxicity, chemokine, naive, inhibitory receptor) were visualized across these three T cell sub-clusters. The results suggested significant changes in gene expressions across four modules after GDNPs treatment (Fig. 5C). Notably, cytotoxicity and chemokine-related genes exhibited higher expression levels in CD8 Teff and Tex sub-clusters with GDNPs treatment.

Furthermore, gene ontology (GO) enrichment analysis indicated that CD8 Teff and CD8 Tex cells, after GDNPs treatment, displayed specific enrichment in genes associated with the cell killing pathway, chemokine production pathway, response to IFN-γ pathway, and T cell activation pathway (Supplementary Fig. 5B, C). In addition, response to IFN-γ pathway, immune receptor activity pathway and MHC protein complex binding pathway were also enriched in naive T (Supplementary Fig. 5D). Using the effector gene set (Table S5) for U Cell analysis, and we found that GDNPs enhanced cytotoxic effector function not only in CD8 Teff, but also in CD8 Tex and naive T (Fig. 5D, E, F). Meanwhile, using M2-related gene set (MRC1, CD163) scores and found that M2-related gene set scores in macrophages after GDNPs treatment were lower (Supplementary Fig. 5D).

Then, we demonstrated the expression of transcription factor Tox, Eomes, and Tbx21 in three T cell sub-clusters using heat maps. The results showed that GDNPs down-regulated Tox, Eomes expression and up-regulated Tbx21 expression in CD8 Teff cells. Same observation was also seen for Tox and Eomes in CD8 Tex (Fig. 5G). Together, these findings indicated that GDNPs enhanced effector function by improving tbx21 expression in CD8 Teff, thereby ameliorating T cell exhaustion.

### The improved effect of GDNPs on T cell exhaustion depended on the mTOR-T-bet axis

**Fig. 6:**
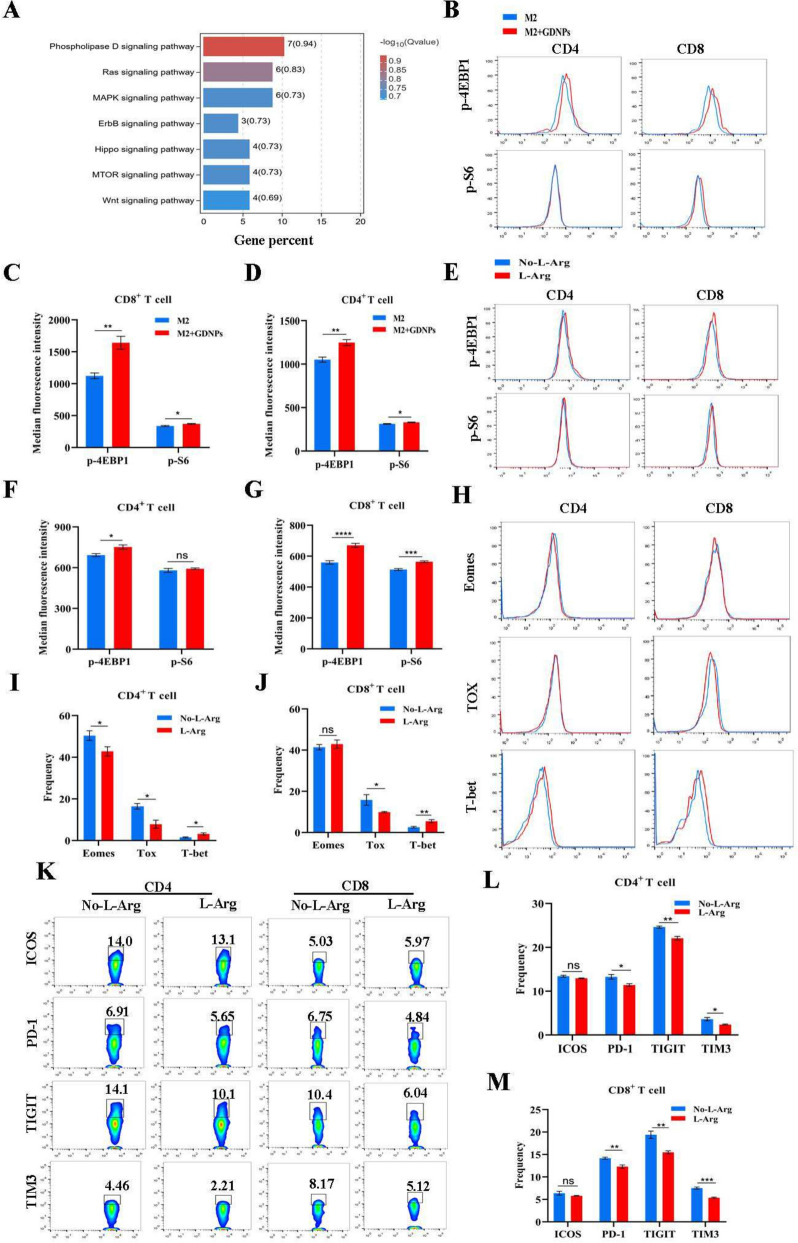
The relieve effect of GDNPs on T cell exhaustion dependent on the mTOR-T-bet axis. **a** KEGG enriched T cells cultured with the supernatant of M2-like macrophages stimulated with or without GDNPs. **b**, **c**, **d** Splenocytes were cultured with M2 supernatant or M2 + GDNPs supernatant for 48 h. Representative histograms and quantification showing the levels of p-4EBP1, p-S6 in CD4 + and CD8 + T cells with or without GDNPs. **e**, **f**, **g** Representative histograms and quantification showing the levels of p-4EBP1, p-S6 in CD4 + and CD8 + T cells with or without L-Arginine. **h**, **i**, **j** Representative histograms and quantification of Emoes, Tox, and T-bet expression in CD4 + and CD8 + T cells with or without L-Arginine. **k**, **l**, **m** Representation of ICOS, PD-1, TITGT and TIM3 expression on CD4 + and CD8 + T cells with or without L-Arginine. The graphs show the frequency of ICOS, PD-1, TITGT, and TIM3 on CD4 + and CD8 + T cells. All results represent the mean ± SEM. Student’s t test (c, d, f, g, i, j, l, m) were used to compare results of different experimental groups for statistically significant difference (*P < 0.05, **P < 0.01, ***P < 0.001, ****P < 0.0001)

Our study demonstrated GDNPs affected arginine level in TME by regulating ARG1 expression. Previously, we found that GDNPs increased the expression of T-bet in T cells, resulting in an improvement in arginine metabolism. This led us to speculate that the amelioration of T cell exhaustion by GDNPs might also be related to the mTOR signaling pathway through the regulation of arginine metabolism. To test whether GDNPs ameliorated T cell exhaustion by regulating mTOR signaling pathway, we constructed a culture system where CD8^+^ T cells were cultured with M2 + GDNPs or the supernatant of M2 macrophages, and the CD8^+^ T cells were harvested 24 h later for transcriptome sequencing. KEGG enrichment analysis of signal transduction showed a significant difference in the mTOR signaling pathway (Fig. 6A). To further explore the mechanism by which GDNPs improved T cell exhaustion, we incubated splenocytes with the supernatant of M2-like macrophages treated with or without GDNPs. Our findings showed that compared to the incubation with supernatant of M2-like macrophages alone, the expression of p-S6 and p-4EBP1 in T cells increased significantly with GDNPs treatment, indicating the enhanced activation of the mTOR signal (Fig. 6B, C, D).

In order to investigate the functional effect of arginine on T cells, we measured the levels of IFN-γ secreted from T cells cultured with different concentration of arginine supplementation. The results showed a positive relationship between the concentration of arginine and the IFN-γ level (Supplementary Fig. 7A). The proliferation assay of T cells suggested that the suppression rates of arginine on CD4^+^ T and CD8^+^ T cells were reduced to approximately 47.45% and 20.6%, respectively (Supplementary Fig. 7B, C, D), compared to the group without L-Arginine. These data confirmed that arginine appreciably promoted T cell activation and proliferation. Notably, arginine is known as an activator of the mTOR pathway and enhances mTOR signal pathway [20]. To further validate the enhancement of mTOR activity by arginine, the flow cytometry results showed that arginine increased the expression of both p-S6 and p-4EBP1 in CD8^+^ T cells but only p-4EBP1 in CD4^+^ T cells (Fig. 6E, F, G).

Furthermore, our flow cytometry data analysis showed that arginine affected certain transcription factors in T cells. The results suggested that arginine decreased the expression of Tox, increased the expression of T-bet in both CD4^+^ T cells and CD8^+^ T cells. While the effect of arginine on Eomes expression was only found in CD4^+^ T cells (down-regulation), but not in CD8^+^ T cells (Fig. 6H, I, J). To test whether arginine supplementation could improve T cell exhaustion by regulating these transcription factors, we detected the expression of ICOS, PD-1, TIGIT, TIM3 on T cells cultured with or without arginine by flow cytometry. Our results showed that supplement of arginine reduced the expression of PD-1, TIGIT and TIM3 on CD4^+^ and CD8^+^ T cells, which confirmed that arginine supplementation ameliorated T cell exhaustion (Fig. 6K, L, M). Therefore, it was plausible that GDNPs improved arginine metabolism by polarizing macrophages, thereby preventing T cell exhaustion.

### Inhibition of mTOR activity in T cells restrain the anti-tumor effect of GDNPs

**Fig. 7:**
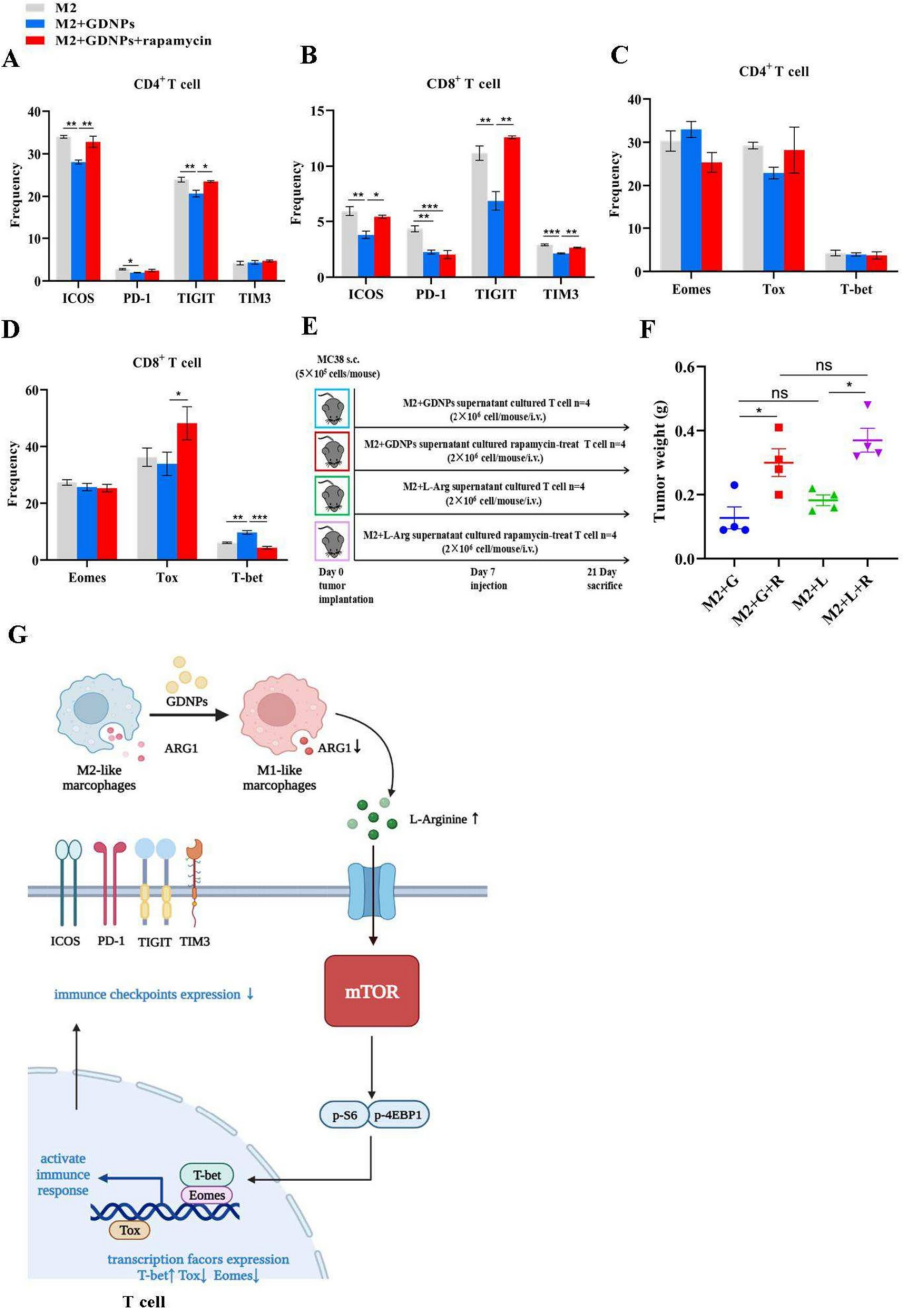
Inhibition of mTOR activity in T cells ineffectively restored the anti-tumor effect of GDNPs. **a**, **b**, **c**, **d** After the monocytes were polarized by M2-like macrophages, M2 supernatants and M2 + GDNPs supernatants with or without Rapamycin (10 nM) were added to incubate splenocytes. **a**, **b** The proportion of ICOS, PD-1, TIGIT, TIM3 on CD4 + and CD8 + T cells in three groups. **c**, **d** The proportion of Eomes, Tox and T-bet in CD4 + and CD8 + T cells in three groups. **e** Schematic illustration of back-transfusion of four T cells into MC38 colon tumor model mice. **f** Tumor weight of M + G, M + G + R, M + L, and M + L + R four groups. **g** The schematic diagram showed the mechanism by which GDNPs relieved T cell exhaustion by reprogramming macrophages. The figure was created using BioRender.com (https://biorender.com). All results represent three independent experiments as the mean ± SEM. One-way ANOVA (a, b, c, d) were used to compare results of different experimental groups for statistically significant difference (*P < 0.05, **P < 0.01, ***P < 0.001)

To further clarify whether GDNPs ameliorated T cell exhaustion through the mTOR-T-bet axis, we used rapamycin, an inhibitor of mTOR activity, to examine whether ameliorated T cell exhaustion was primarily regulated by mTOR signaling pathway. Thus, we established splenocyte culture system using the supernatant of M2-like macrophages with GDNPs plus rapamycin to inhibit mTOR activity. FACS analysis of p-S6 and p-4EBP1 showed that rapamycin inhibited mTOR activity in T cells (Supplementary Fig. 7A, B, C). The flow cytometry results showed that the expression ratios of ICOS, PD-1, TIGIT on T cells were significantly higher when rapamycin was added in comparison to M2 + GDNPs alone (Fig. 7A, B). Note that this difference was observed for TIM3 only in CD8^+^ T cells (Fig. 7B). Tox expression was higher in CD8^+^ T cells treated with rapamycin compared to the group without rapamycin, while T-bet expression was lower with the presence of rapamycin (Fig. 7C, D). Meanwhile, we conducted validation in vivo, T cells treated with or without rapamycin were cultured from the supernatant of M2-like macrophages that were either stimulated by GDNPs or not, or of M2-like macrophages with or without arginine for. Then, these T cells were transfused into MC38-bearing mice model (Fig. 7E). The results showed that GDNPs lost its anti-tumor effect with rapamycin treatment (Fig. 7F, Supplementary Fig. 8A). After the transfusion of rapamycin-treated T cells, mTOR activity of T cells in TME was inhibited (Supplementary Fig. 8B, C) and the expression of transcription factor T-bet was decreased (Supplementary Fig. 8D, E). In summary, the effect of GDNPs to ameliorate T cell exhaustion and inhibit tumor growth was weakened in the presence of rapamycin, demonstrating a dependence on the mTOR-T-bet pathway for their efficacy (Fig. 7G).

## Discussion

T cell exhaustion is a functional deficiency of T cells during chronic infection and cancer [21]. It was defined as poor effector function, sustained expression of inhibitory receptors, and a transcriptional state distinct from effector T cells, resulting in an inability to secrete cytokines in response to antigen stimulation [22]. In recent years, the mechanisms of T cell exhaustion have been extensively studied in cancer research as well as chronic infection, and autoimmune diseases [23, 24].

Currently, inhibitors of PD-1 and other immune checkpoints have been used in tumor immunotherapy to successfully reduce T cell exhaustion [25, 26]. For example, PD-1 blocking therapy was found to reactivate and improve the metabolic function of partially exhausted CD8^+^ T cells [27, 28]. However, many tumors exhibit limited responsiveness to immune checkpoint inhibitors, impeding productive anti-tumor immune responses [29, 30]. This observation may be related to the regulatory exhaustion of specific transcription factors [31, 32]. Therefore, revealing the mechanism by which T cells regulated exhaustion through transcription factors could improve the efficacy of immune checkpoint inhibitors.

TAMs are one of the most abundant immune cells in TME, supporting tumor growth, angiogenesis, invasion and metastasis [33, 34]. Thus, TAMs have been regarded as promising targets for novel anticancer agents [35]. In general, TAMs, including tumoricidal M1 and tumor-supportive M2 macrophages, are highly plastic and exhibit opposing phenotypes and functions. They played key roles in tumor progression but can also contribute to anti-tumor immunity [36]. M2-like macrophages produce ARG1 and other anti-inflammatory factors to promote tumor, while M1-like macrophages produce TNF-α and other pro-inflammatory factors to inhibit tumor [37, 38]. Macrophages with M2-like phenotype are predominant in most of tumor types. Shifting the M1/M2 ratio towards a M1-like phenotype has emerged as attractive therapeutic strategies in cancer treatment.

Our previous studies demonstrated that GDNPs, a novel EVs-liked derived from ginseng [11], increased T cell infiltration and activated T cells in the TME of the colorectal cancer bearing mice by reprogramming TAMs. GDNPs combined with PD-1 reduced the expressions of immune checkpoints (TIM3, PD-1 and ICOS). Moreover, GDNPs alone also effectively inhibited the expression of immune checkpoints, but the mechanism remained unclear [12]. Therefore, our aim was to explore whether GDNPs could ameliorate T cell exhaustion by reprogramming TAMs and reducing T cell immune checkpoints.

It has been reported that ARG1 is widely present in tumors, and the increased activity is associated with advanced disease and poor clinical prognosis [39]. ARG1 degrades arginine which is essential in T cell activation and proliferation [40]. Thus, an increased ARG1 expression is a key factor for the rapid growth and deterioration of tumor [41]. We had proved that GDNPs reduced ARG1 expression by polarization of macrophages. It has been confirmed that GDNPs reprogrammed TAMs to down-regulate ARG1 expression [11]. Therefore, we performed a preliminary study to evaluate whether GDNPs reducing ARG1 from TAMs with GDNPs treatment could decrease expression of immune checkpoints. Firstly, through bioinformatics, the data suggested that ARG1 expression was positively correlated with immune checkpoints in colorectal cancer and other tumors. Next, single cell analysis showed that ARG1 was principally derived from MPs, especially TAMs. ARG1 enhanced immune checkpoints expression and promoted T cell exhaustion by degrading arginine. Next, we explored how to reduce ARG1 expression and reverse T cell exhaustion. Based on the above research basis and background, GDNPs could reduce ARG1 expression by polarizing M2-like macrophages, which might be more conducive to inhibiting ARG1 production and maintaining arginine level in TME. To clarify this, M2-like macrophages—the dominant TAMs in colon tumor model in our previous study were used to simulate TAMs. The M2-like macrophages were treated with GDNPs and we found that GDNPs could reprogram macrophages to reduce ARG1 expression. In addition, GDNPs inhibited ARG1 secretion from M2-like macrophages and significantly improved arginine level. These above findings suggested that GDNPs improved arginine level by reprogramming macrophages to reduce ARG1 production.

In addition, we further explored how GDNPs ameliorated T cell exhaustion through arginine metabolism. Arginine has been reported to encourage T cells to develop an immune response [42]. ARG1 could inhibit T cell activation and proliferation, leading to T cells dysfunction and allowing tumor cells to escape [43, 44]. Nevertheless, whether ARG1 regulates T cell exhaustion has not been reported. Immune-checkpoint blockade (ICB) targeting the PD-1/PD-L1 pathway mediates durable remissions in a subset of cancer patients, with these effects generally attributed to the reversal of CD8^+^ T cell exhaustion in tumor microenvironment (TME). However, not all exhausted CD8^+^ T cells can respond to PD-1/PD-L1 ICB [45]. How to reverse or ameliorate T cell exhaustion remains a challenge to improve tumor immunotherapy. Therefore, we investigated the function of T cells cultured with reduced ARG1 production by GDNPs polarizing macrophages. First of all, arginine level was maintained to some extent in the culture system after GDNPs treatment. Secondly, inhibition of ARG1 production in the supernatant of M2-like macrophages or addition of arginine resulted in improving T cells activation and proliferation. Meanwhile, GDNPs reduced ARG1 production by polarizing macrophages, improving arginine metabolism to promote T cell activation and proliferation. We further investigate whether GDNPs also reversed T cell exhaustion through promoting T cell effector function. Single-cell sequencing analysis once again confirmed that GDNPs could up-regulate T-bet expression in CD8 Teff to enhance its effecting function and reduce Tox, Eomes expression in CD8 Tex to improve its exhaustion state, thereby ameliorating T cell exhaustion. In this study, we found that CD8^+^ T cells were activated with enhanced the cytotoxic activity in CD8 Teff and chemokine activity in CD8 Tex, but the absolute number of CD8 Tex cells did not significantly decreased. Furthermore, in the process of exploring T cell exhaustion reversion, discovering that GDNPs reduced the expression of ICOS, PD-1, TIGIT, TIM3 and transcription factors Eomes and Tox, with up-regulation of transcription factor T-bet. These data indicated that arginine activity in TME was affected by the concentration of ARG1, which regulated T cell activation, proliferation and exhaustion. Finally, in vivo experiments showed that GDNPs reprogrammed TAMs to reduce ARG1 expression and improve arginine metabolic pathways in TME, thereby activating T-bet to inhibit immune checkpoints expression and ameliorate T cell exhaustion.

Then, the mammalian target of rapamycin (mTOR) signaling pathway in T cells is one of the important arginine-mediated pathways, playing an important role in T cell activation and proliferation [46, 47]. In inflammation-related diseases including malignant tumors, abnormal expression or activity change of protein kinases induce abnormal activation of related signaling pathways and dysfunction [48]. mTOR regulates the body's immune response by promoting phosphorylation of ribosomal protein S6 kinase β-1 (S6K1) and eukaryotic translation initiation factor 4E-binding protein 1 (4E-BP1) and other targets. S6 is activated from p-S6K1 to p-S6 [49]. Therefore, p-S6 and p-4EBP1 can be the important indicators of mTOR activity enhancement [50]. Although the effect of arginine on the mTOR pathway has been already investigated [51], our results demonstrated that GDNPs slowed the degradation of arginine level in TME by polarizing TAMs, and arginine could enhance mTOR activity to improve the immune function of T cells. Recent studies suggest that the enhancing mTOR activity may improve the effect of T cells by up-regulating T-bet [52, 53]. Our flow cytometry analysis of transcription factors showed that GDNPs ameliorating T cell exhaustion were related to the transcription factor T-bet in T cells. Combining with the background knowledge, we speculated that the expression of T-bet was regulated by mTOR to promote T cell effector function [54, 55]. Besides, mTOR activity in T cells by flow cytometry was detected and the result suggested that GDNPs enhanced mTOR activity. This might be closely related to the regulation of arginine metabolism by GDNPs. So, we supposed that GDNPs could ameliorate T cell exhaustion by regulating the mTOR signaling pathway through improving arginine metabolism. Subsequently, we found that arginine activated mTOR to up-regulate T-bet expression and down-regulate Eomes and Tox expression to decrease immune checkpoints expression. To further clarify that GDNPs relied on the mTOR-T-bet pathway to ameliorate T cell exhaustion, we used rapamycin to inhibit mTOR activity for reverse verification. By inhibiting mTOR activity, GDNPs could not effectively up-regulate T-bet to reduce immune checkpoint expression, even when arginine levels were still relatively high. To sum up, GDNPs reprogrammed TAMs to decrease ARG1 production, which improved arginine metabolism, ultimately enhancing mTOR-T-bet to ameliorate T cell exhaustion.

## Conclusion

Together, our data showed that GDNPs could ameliorate T cell exhaustion and also discovered the mechanism of such process (Fig. 7G). GDNPs effectively reprogrammed TAMs to polarize M2-like macrophages into M1-like macrophages, and exerting anti-tumor immune response with T cell activation, thus significantly inhibiting the growth of colorectal cancer in mice. The negative regulatory factor ARG1 in TME was remarkably reduced, improving the arginine metabolic pathway and reshaping the immunosuppressive microenvironment to some extent. Because GDNPs altered ARG1 expression, arginine level was elevated to activate mTOR activity, along with the up-regulation of T-bet expression regulated by mTOR activity. While the expression of transcription factors Eomes and Tox in T cells promoting T cell exhaustion was inhibited, ultimately reducing the expression of immune checkpoint and ameliorating T cell exhaustion. Our research found that the nanovesicles of the medicinal plant ginseng could ameliorate T cell exhaustion through this mechanism, restoring the anti-tumor effect of T cells and better treating colorectal cancer. Meanwhile, the research also provided new strategies to better promote immune checkpoint inhibitors to treat tumors in this way.

### Supplementary Information

**Additional file 1.** Supplemental materials and methods [56–60].

**Additional file 2:** **Figure S1.** ARG1 was mainly derived from tumor-associated macrophages. a The expression of ARG1 in colorectal cancer patients was analyzed using the GSE4107 dataset. b Arginase1^+^ cells, macrophages (F4/80^+^) were detected in fixed colon tumor serial sections using immunofluorescence. Bar=20 μm. c ARG1 expression in tumor cells (CD45^-^ cells) and immune cells (CD45^+^ cells) determined by flow cytometry. Orange represents isotype control, blue reflects ARG1 expression of CD45^-^ cells, and red reflects ARG1 expression of CD45^+^ cells. d Frequency of main immune cells infiltrated in MC38 tumor. e Pie chart of the proportion of Macrophages, MDSC, DCs cells, T cells, NK cells, and other immune cells in mouse MC38 tumor environment. f The proportion of ARG1 expression in the main immune cell. **Figure S2.** GDNPs decreased ARG1 expression in M2-like macrophages. a ARG1/GAPDH ratios. **Figure S3.** GDNPs could effectively inhibit the growth of MC38 colon tumor in mice. a The tumor size of Vehicle and GDNPs group was photographed. b Tumor weights of two groups. c Body weights of two groups. All results represent the mean ± SEM (*n*=5). Two-way ANOVA (c) and Student’s t test (b) were used to compare results of different experimental groups for statistically significant difference (***P < 0.001). **Figure S4.** GDNPs could regulate the expression of immune checkpoints and transcription factors. a Representative histograms of ICOS, PD-1, TIGIT, TIM3 on CD4+ and CD8^+^ T cells in M2, M2+GDNPs, M2+nor-NOHA and M2+L-Arg groups. b Representative histograms of Emoes, Tox, and T-bet in CD4^+^ and CD8^+^ T cells in M2, M2+ GDNPs, M2+ nor-NOHA and M2+ L-Arg groups. The results represent three independent experiments as the mean ± SEM. **Figure S5.** GDNPs promoted the immune effector function of CD8 Teff. a The proportion of immune cells in MC38 colon cancer of mice was analyzed by single cell sequencing. b GO enrichment analysis was performed in CD8 Teff (G VS V). c GO enrichment analysis was performed in CD8 Tex (G VS V). d GO enrichment analysis was performed in naive T (G VS V). e Ucell score was performed for gene sets with M2 polarization characteristics in macrophages. Wilcox (e) was used to compare results of different experimental groups for statistically significant difference  (****P < 0.0001). **Figure S6.** L-Arginine promoted T cell activation and proliferation. a L-Arginine depletion prevented IFN-γ secretion by splenocytes. Splenocytes were activated with αCD3/CD28 in the presence of media with L-Arginine levels of 0, 5, 50, 100, 1000 μM. After 48 h, IFN-γ was determined by ELISA in the culture supernatants. b, c, d T cells proliferation assay. Splenocytes were cultured in medium with and without L-Arginine. The proliferation of CD4^+^ and CD8^+^ T cells was detected by Flow cytometry. The results represent three independent experiments as the mean± SEM. Student’s t test (a, c, d) were used to compare results of different experimental groups for statistically significant difference (*P < 0.05, **P < 0.01). **Figure S7.** Rapamycin inhibited mTOR activity in GDNPs-acting T cells a, b, c Rapamycin (10 nM) was added or not added to M2+GDNPs supernatants to incubate splenocytes. Representative histograms and quantification showing the levels of p-4EBP1, p-S6 in CD4^+^ and CD8^+^ T cells. The results represent three independent experiments as the mean ± SEM. Student’s t test (b, c) were used to compare results of different experimental groups for statistically significant difference (**P < 0.01, ***P < 0.001, ****P < 0.0001). **Figure S8.** Inhibition of mTOR activity ineffectively restored the anti-tumor effect of GDNPs. a The tumor size of M2+G, M2+G+R, M2+L and M2+L+R four groups was photographed (*n*=4). b, c Flow cytometry was used to detect the expression of p-4EBP1 and p-S6 in T cells from four groups. d, e The transcription factor T- bet in T cells from four groups was detected by flow cytometry. One-way ANOVA (b, c, d, e) was used to compare results of different experimental groups for statistically significant difference (*P < 0.05, **P < 0.01, ***P < 0.001, ****P < 0.0001). **Figure S9.** Representative gating strategies for flow cytometry. **Figure S10.** Unprocessed original images of gels and western blots.

**Additional file 3:** **Table S1.** Antibodies used for flow cytometry, western blot. **Table S2.** Drug information. **Table S3.** Experimental Models: Cell Lines. **Table S4.** Primer sequences for real-time RT-PCR analysis. **Table S5.** Effector function gene set.

**Additional file 4:** **Table S6.**

## Data Availability

The datasets analyzed during the current study are available in in this study and its Supplementary materials, or are available from the corresponding author (P. Cao and M. Cao) on reasonable request. The processed gene expression data of this study can be obtained from Gene Expression Omnibus (GEO) with an accession number of GSE241285.

## Abbreviations

ICs: Immune checkpoints
GDNPs: Ginseng-derived nanoparticles
TAMs: Tumor-associated macrophages
TME: Tumor microenvironment
ARG1: Arginase-1
BRCA: Breast invasive carcinoma
CHOL: Cholangiocarcinoma
COAD: Colon adenocarcinoma
HSNC: Head and neck squamous cell carcinoma
KIRP: Kidney renal papillary cell carcinoma
LIHC: Liver hepatocellular carcinoma
LUSC: Lung squamous cell carcinoma
SKCM: Skin cutaneous Melanoma
STAD: Stomach adenocarcinoma
THCA: Thyroid carcinoma
UCEC: Uterine corpus endometrial carcinoma
LUAD: Lung adenocarcinoma
PAAD: Pancreatic adenocarcinoma
PCPG: Pheochromocytoma and paraganglioma
THYM: Thymoma
LGG: Brain lower grade glioma
MESO: Mesothelioma
PRAD: Prostate adenocarcinom
TGCT: Testicular germ cell tumors
L-Arg: L-Arginine
PD-1: Programmed cell death protein 1
TIM3: T cell immunoglobulin and mucin domain-containing protein 3
TIGIT: T cell immunoreceptor with Ig and ITIM domains
ICOS: Inducible T cell co-stimulator
mTOR: Mammalian target of rapamycin
nor-NOHA: Nω-hydroxy-nor-Arginine
