# Supplementary material for: Ginseng-derived nanoparticles reprogram macrophages to regulate arginase-1 release for ameliorating T cell exhaustion in tumor microenvironment
Source: J Exp Clin Cancer Res. 2023 Nov 28;42:322. doi: 10.1186/s13046-023-02888-7 (PMC10683135; doi:10.1186/s13046-023-02888-7)
Supplement: Supplementary file 1 — Additional file 1. Supplemental materials and methods [56–60]. [file 13046_2023_2888_MOESM1_ESM.doc]

**Supplemental materials and methods**

**GDNPs preparation and characterization**

The preparation and identification of GDNPs were based on the previous method [11, 12].

**RNA isolations, quantitative real-time (q) PCR**

Total RNA was isolated from samples using Trizol reagent (TaKaRa, Japan) and reverse transcribed into cDNA using a cDNA Synthesis Kit (TaKaRa, Japan) according to the manufacturer’s protocol. Then, RT-PCR was performed using SYBR Green Mastermix (Toyobo, Japan) following the manufacturer’s instructions and run on an ABI Prism 7500 Sequence Detection System (Applied Biosystems, USA). The primer sequences are shown in Supplementary Table S4. The 2ΔΔCt method was used to calculate fold changes in gene expression normalized to the housekeeping gene Gapdh (Supplementary Table S4).

**Western blotting**

Lysed cells were boiled in Laemmli loading buffer, separated using SDS–PAGE and transferred to PVDF membrane by semi-dry blotting. After blocking with 5% BSA in Tris-buffered saline/1% Tween-20 membranes were probed with primary antibodies followed by incubation with appropriate HRP-conjugated secondary antibodies. Antibodies used for detection are listed in Supplementary Table S1. Odyssey Infrared Imaging System from LI-COR were used for image acquisition. Image J was used for densitometric analysis of the blots.

**Library preparation and scRNA-seq**

Single-cell suspensions (1×10^5^ cells/mL) with PBS were loaded into microfluidic devices using the Singleron Matrix® Single Cell Processing System (Singleron). Subsequently, the scRNA-seq libraries were constructed according to the protocol of the GEXSCOPE® Single Cell RNA Library Kits (Singleron) [56]. Individual libraries were diluted to 4 nM and pooled for sequencing. At last, pools were sequenced on Illumina HiSeq X with 150 bp paired end reads.

**scRNA-seq quantifications and statistical analysis**

Briefly, after removing low-quality reads, cell barcode and UMI were extracted. Reads were mapped to the mice genome reference sequence GRCm38 with ensemble version 92 gene annotation [57]. The UMI count tables of each cellular barcode were employed for further analysis. Cells were filtered according to the following criteria: gene count below 200, top 2% gene counts, and top 2% UMI counts. Cells with over 20% mitochondrial content were removed. Cell type identification and clustering analysis were performed by Seurat program (http://satijalab.org/seurat/, R package,v.3.0.1) [58, 59]. Afterwards, parameter resolution to 1.2 was set for FindClusters function to clustering analyses.

**Spatial metabolomics assay**

The tumors were fixed at −80℃ with optimal cutting temperature compound (OCT; Sakura Finetek, Torrance, CA, United States). The tumor were cut into consecutive sagittal slices 10 μm about 10 slices by a cryostat microtome (Leica CM 1950, Leica Microsystem, Germany) and were thaw-mounted on positive charge desorption plate (Thermo Scientific, U.S.A). Then, they were left for hematoxylin-eosin (H&E) staining.

The analyses were carried out with an AFADESI-MSI platform (Beijing Victor Technology Co., LTD, Beijing, China) in tandem with a Q-Orbitrap mass spectrometer (Q Exactive, Thermo Scientific, U.S.A.). For more details as reported [23]. Here, a mixture of acetonitrile and water (8:2, v/v, 5 μL/min) was used as spray solvent, the transporting gas flow rate was 45 L/min and the spray voltage was set at 7 kV. The MS resolution was set at 70,000, the mass range was 70-1000 Da, the automated gain control (AGC) target was 2E6, the maximum injection time was set to 200 ms, the S-lens voltage was 55 V, and the capillary temperature was 350°C. The MSI experiment was carried out with a constant rate of 0.2 mm/s continuously scanning the surface of tumor section in the x direction and a 100 μm vertical step in the y direction.

AFADESI–MSI data analysis was been described [60]. In brief, convert the collected data into the corresponding format and the data imported into MSiReader (an open-source interface to view and analyze high resolving power MS imaging files on Matlab platform) for ion image reconstructions. After background subtraction, region-specific MS profiles were precisely extracted by matching high-spatial resolution H&E images. Then, the relative intensities of ions were performed to acquire histology-specific discriminating metabolites by using OPLS-DA. To verify whether the metabolites of difference between groups were significant, a two-tailed Student’s T-test was used. Differential metabolites were selected with VIP values greater than 1.0 and p-values less than 0.05.

**Assay of arginine on T cells**

IFN-γ secretion was stimulated by treatment with anti-CD3 (0.5 μg/mL) and anti-CD28 (0.5 μg/mL). Splenocytes were cultured in medium with 0μm, 5μm, 50μm, 100μm, 1000μm L-Arginine. After 48 h, the supernatant was collected and tested.

Splenocytes were labeled with Cell Trace CFSE dye (2 μM final concentration, Thermo Fisher Scientific) according to manufacturer’s manual. The labeled splenocytes were plated in round-bottomed 24-well plates (1–2×10^6^/well) in L-Arginine-free RPMI-medium or with L-Arginine (1 mM) and stimulated with anti-CD3/CD28 (0.5 μg/mL) (Table S2). After incubation for 72 h at 37°C, 5% CO_2_ splenocytes were harvested, stained with anti-CD3-APC/Cy7, anti-CD4-APC, or anti-CD8-BV421 (Table S1) and analyzed by FACS Aria II system (BD Biosciences, USA).

Splenocytes were cultured in medium with or without L-Arginine (1 mM). After 48 h, the supernatant was collected to detect mTOR activity and transcription factors in T cells and exhaustion indicators T cell.
