# Supplementary material for: Ginseng-derived nanoparticles reprogram macrophages to regulate arginase-1 release for ameliorating T cell exhaustion in tumor microenvironment
Source: J Exp Clin Cancer Res. 2023 Nov 28;42:322. doi: 10.1186/s13046-023-02888-7 (PMC10683135; doi:10.1186/s13046-023-02888-7)
Supplement: Supplementary file 2 — Additional file 2: Figure S1. ARG1 was mainly derived from tumor-associated macrophages. a The expression of ARG1 in colorectal cancer patients was analyzed using the GSE4107 dataset. b Arginase1+ cells, macrophages (F4/80+) were detected in fixed colon tumor serial sections using immunofluorescence. Bar=20 μm. c ARG1 expression in tumor cells (CD45- cells) and immune cells (CD45+ cells) determined by flow cytometry. Orange represents isotype control, blue reflects ARG1 expression of CD45- cells, and red reflects ARG1 expression of CD45+ cells. d Frequency of main immune cells infiltrated in MC38 tumor. e Pie chart of the proportion of Macrophages, MDSC, DCs cells, T cells, NK cells, and other immune cells in mouse MC38 tumor environment. f The proportion of ARG1 expression in the main immune cell. Figure S2. GDNPs decreased ARG1 expression in M2-like macrophages. a ARG1/GAPDH ratios. Figure S3. GDNPs could effectively inhibit the growth of MC38 colon tumor in mice. a The tumor size of Vehicle and GDNPs group was photographed. b Tumor weights of two groups. c Body weights of two groups. All results represent the mean ± SEM (n=5). Two-way ANOVA (c) and Student’s t test (b) were used to compare results of different experimental groups for statistically significant difference (***P < 0.001). Figure S4. GDNPs could regulate the expression of immune checkpoints and transcription factors. a Representative histograms of ICOS, PD-1, TIGIT, TIM3 on CD4+ and CD8+ T cells in M2, M2+GDNPs, M2+nor-NOHA and M2+L-Arg groups. b Representative histograms of Emoes, Tox, and T-bet in CD4+ and CD8+ T cells in M2, M2+ GDNPs, M2+ nor-NOHA and M2+ L-Arg groups. The results represent three independent experiments as the mean ± SEM. Figure S5. GDNPs promoted the immune effector function of CD8 Teff. a The proportion of immune cells in MC38 colon cancer of mice was analyzed by single cell sequencing. b GO enrichment analysis was performed in CD8 Teff (G VS V). c GO enrichment analysis was pe [file 13046_2023_2888_MOESM2_ESM.pdf]

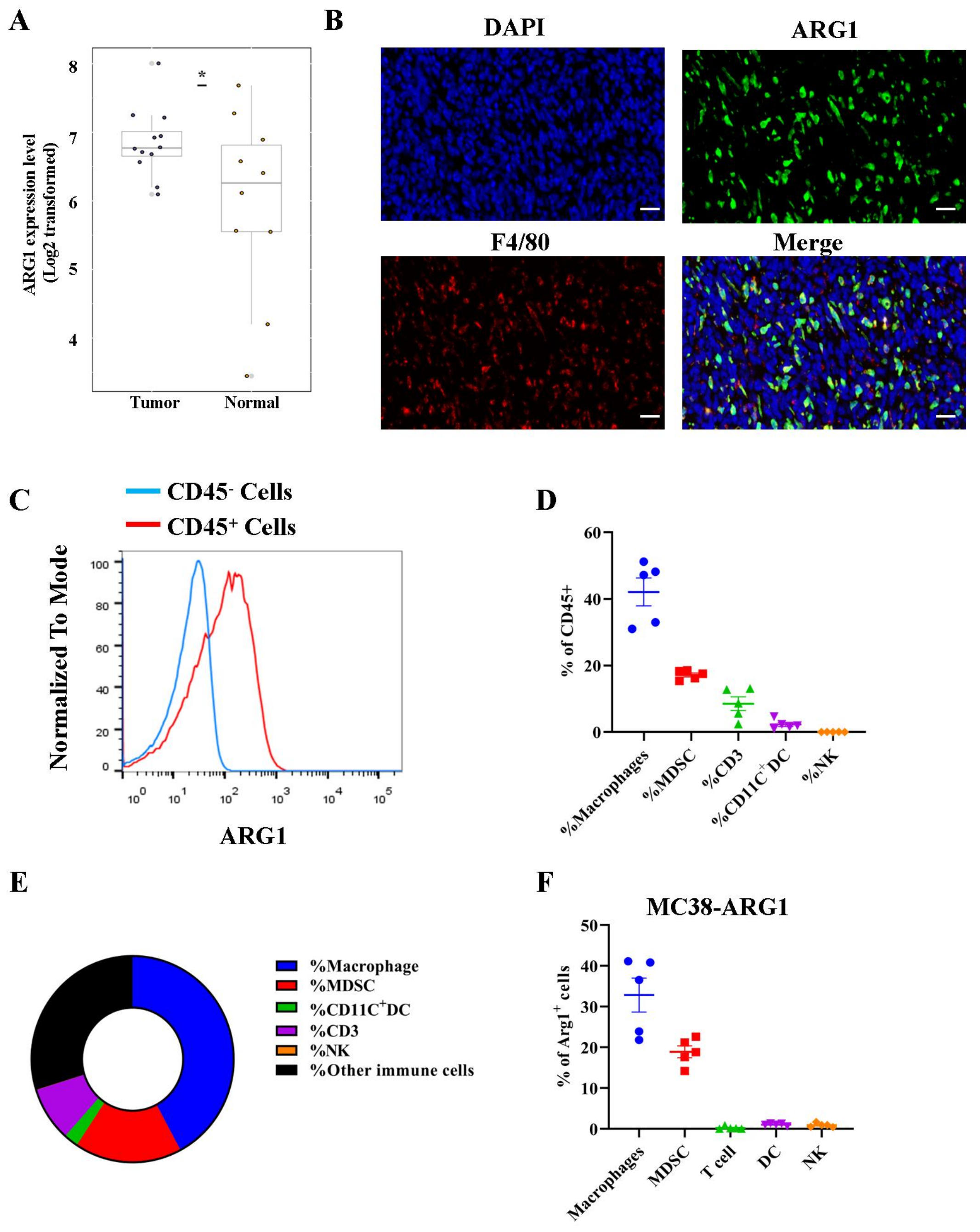

Fgure. S1. ARG1 was mainly derived from tumor-associated macrophages. **a** The expression of ARG1 in colorectal cancer patients was analyzed using the GSE4107 dataset. **b** Arginase1^{+} cells, macrophages (F4/80^{+}) were detected in fixed colon tumor serial sections using immunofluorescence. Bar=20 μm. **c** ARG1 expression in tumor cells (CD45^{-} cells) and immune cells (CD45^{+} cells) determined by flow cytometry. Orange represents isotype control, blue reflects ARG1 expression of CD45^{-} cells, and red reflects ARG1 expression of CD45^{+} cells. **d** Frequency

of main immune cells infiltrated in MC38 tumor. **e** Pie chart of the proportion of Macrophages, MDSC, DCs cells, T cells, NK cells, and other immune cells in mouse MC38 tumor environment. **f** The proportion of ARG1 expression in the main immune cell.

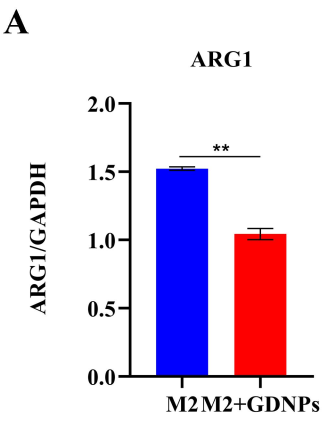

Figure. S2. GDNPs decreased ARG1 expression in M2-like macrophages. **a** ARG1/GAPDH ratios.

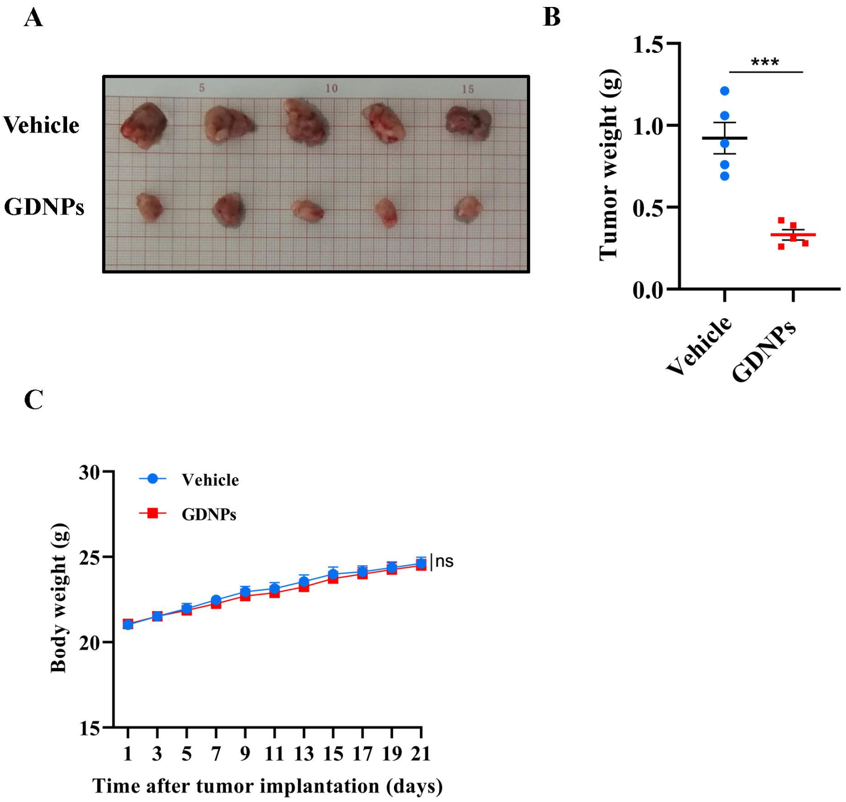

Figure. S3. GDNPs could effectively inhibit the growth of MC38 colon tumor in mice. **a** The tumor size of Vehicle and GDNPs group was photographed. **b** Tumor weights of two groups. **c** Body weights of two groups. All results represent the mean± SEM (n=5). Two-way ANOVA (c)

and Student's t test (b) were used to compare results of different experimental groups for statistically significant difference ($^{***}P < 0.001$).

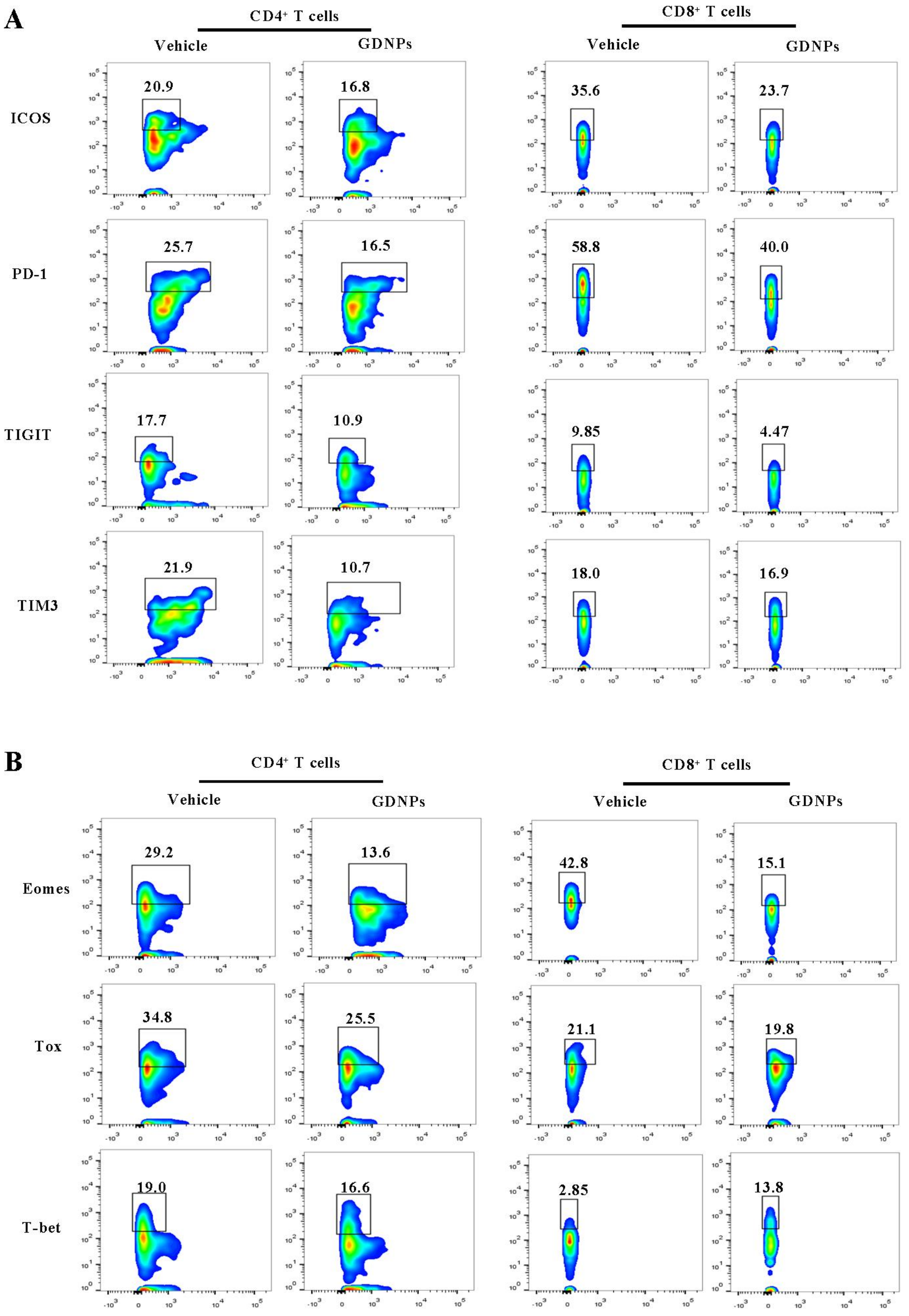

Figure. S4. GDNPs could regulate the expression of immune checkpoints and transcription factors. **a** Representative histograms of ICOS, PD-1, TIGIT, TIM3 on $CD4^{+}$ and $CD8^{+}$ T cells in M2,

M2+GDNPs, M2+nor-NOHA and M2+L-Arg groups. **b** Representative histograms of Emoes, Tox, and T-bet in $CD4^{+}$ and $CD8^{+}$ T cells in M2, M2+ GDNPs, M2+ nor-NOHA and M2+ L-Arg groups. The results represent three independent experiments as the mean ± SEM.

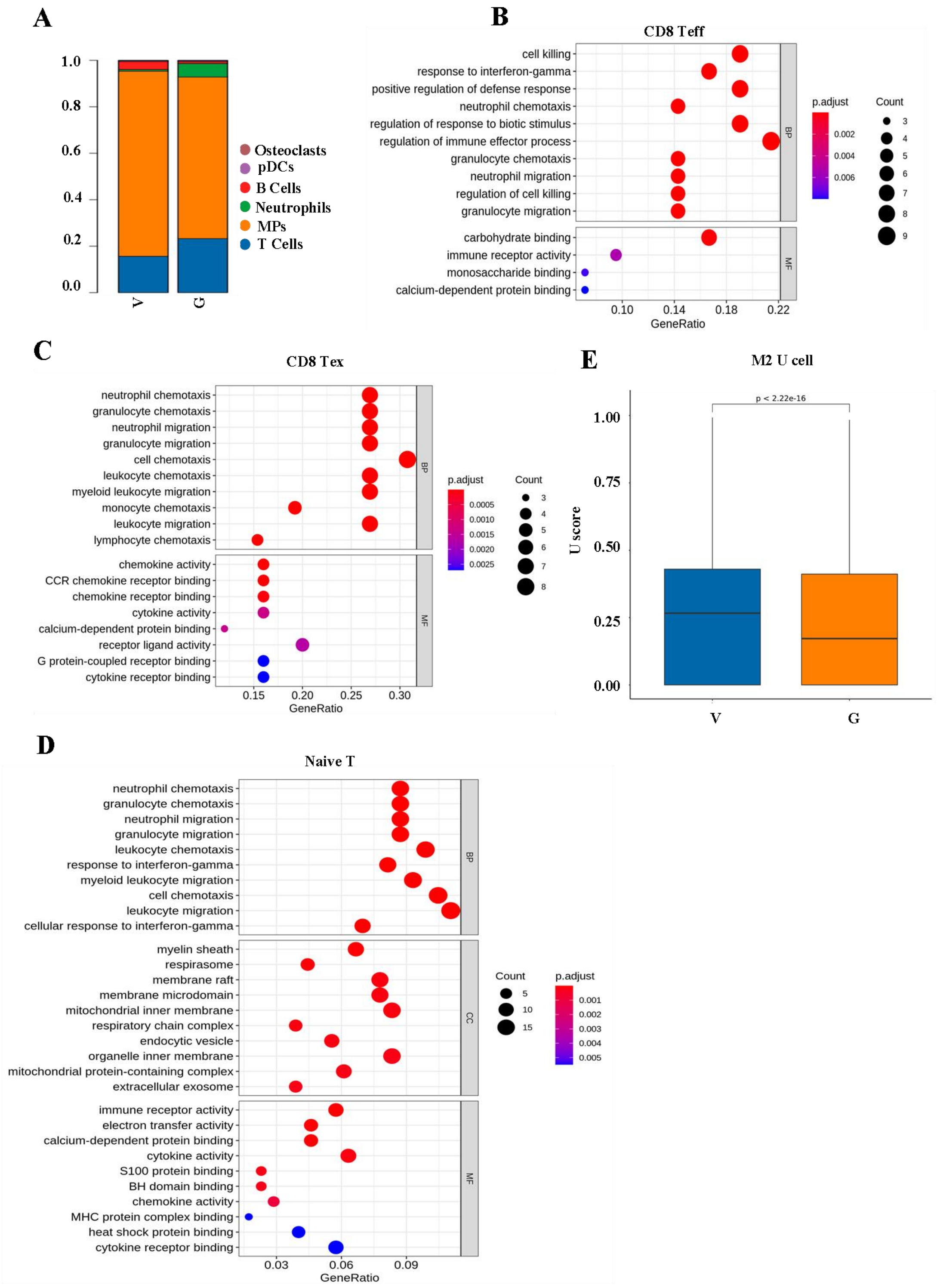

Figure. S5. GDNPs promoted the immune effector function of CD8 Teff. **a** The proportion of immune cells in MC38 colon cancer of mice was analyzed by single cell sequencing. **b** GO enrichment analysis was performed in CD8Teff (G VS V). **c** GO enrichment analysis was performed in CD8 Tex (G VS V). **d** GO enrichment analysis was performed in naive T (G VS V). **e** Ucell score was performed for gene sets with M2 polarization characteristics in macrophages. Wilcox (e) was used to compare results of different experimental groups for statistically significant difference ($^{****}P < 0.0001$).

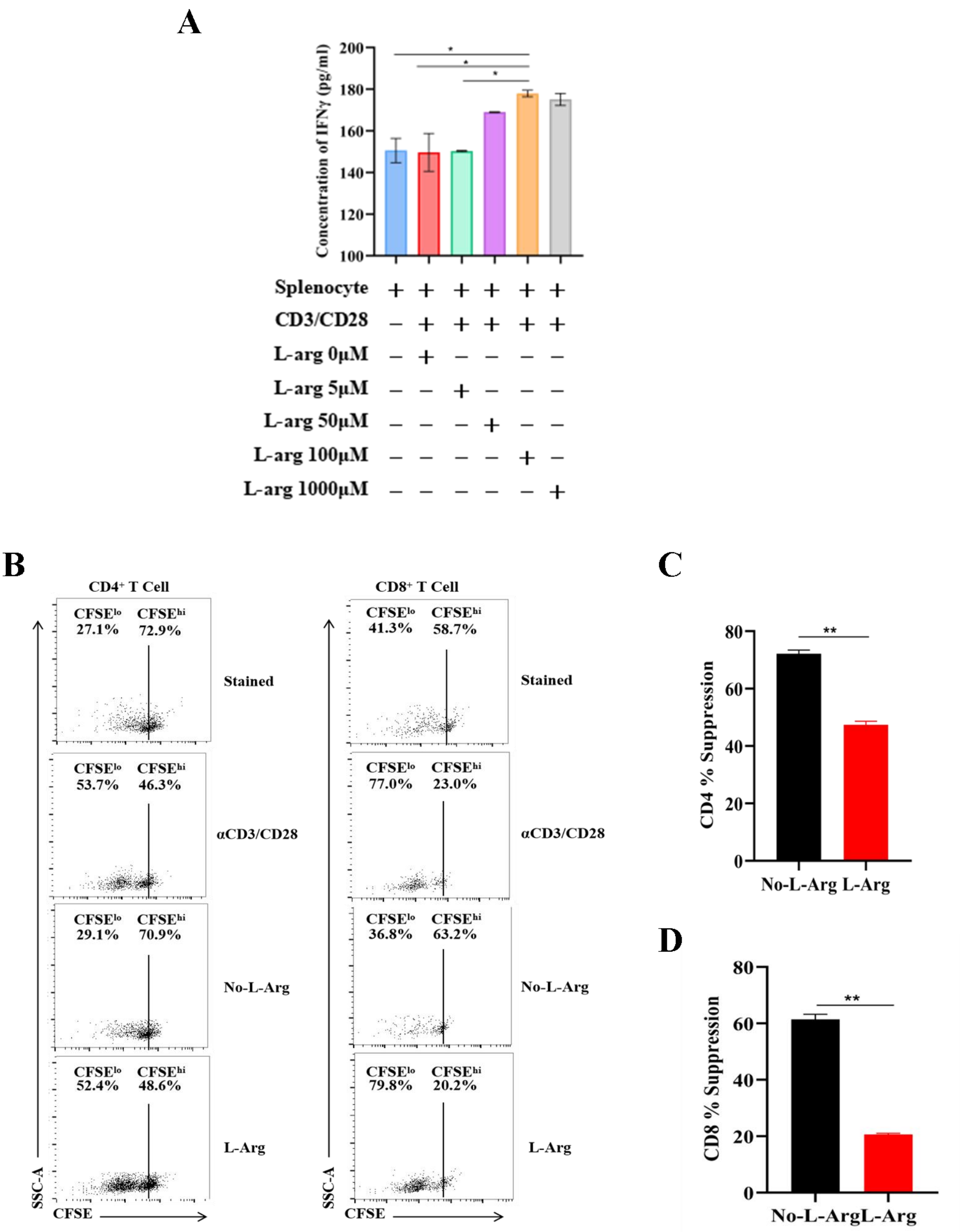

Figure. S6. L-Arginine promoted T cell activation and proliferation. **a** L-Arginine depletion prevented IFN-γ secretion by splenocytes. Splenocytes were activated with αCD3/CD28 in the presence of media with L-Arginine levels of 0, 5, 50, 100, 1000 μM. After 48 h, IFN-γ was determined by ELISA in the culture supernatants. **b, c, d** T cells proliferation assay. Splenocytes were cultured in medium with and without L-Arginine. The proliferation of $CD4^+$ and $CD8^+$ T cells was detected by Flow cytometry. The results represent three independent experiments as the

mean ± SEM. Student's t test (a, c, d) were used to compare results of different experimental groups for statistically significant difference ($^{*}P < 0.05$, $^{**}P < 0.01$).

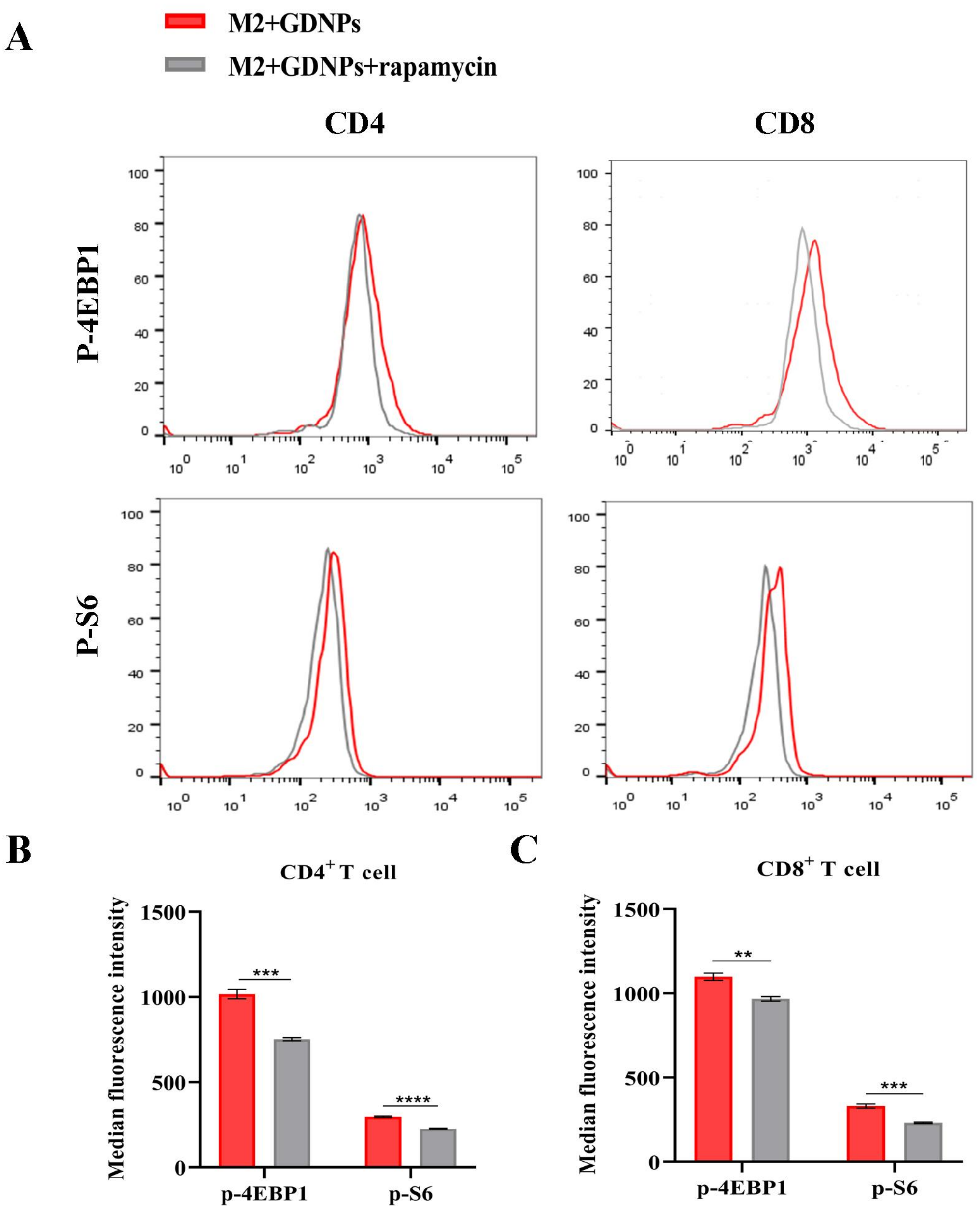

Figure. S7. Rapamycin inhibited mTOR activity in GDNPs-acting T cells **a, b, c** Rapamycin (10nM) was added or not added to M2+GDNPs supernatants to incubate splenocytes. Representative histograms and quantification showing the levels of p-4EBP1, p-S6 in $CD4^{+}$ and $CD8^{+}$ T cells. The results represent three independent experiments as the mean ± SEM. Student's t test (b, c) were used to compare results of different experimental groups for statistically significant

difference ($^{**}P < 0.01$, $^{***}P < 0.001$, $^{****}P < 0.0001$).

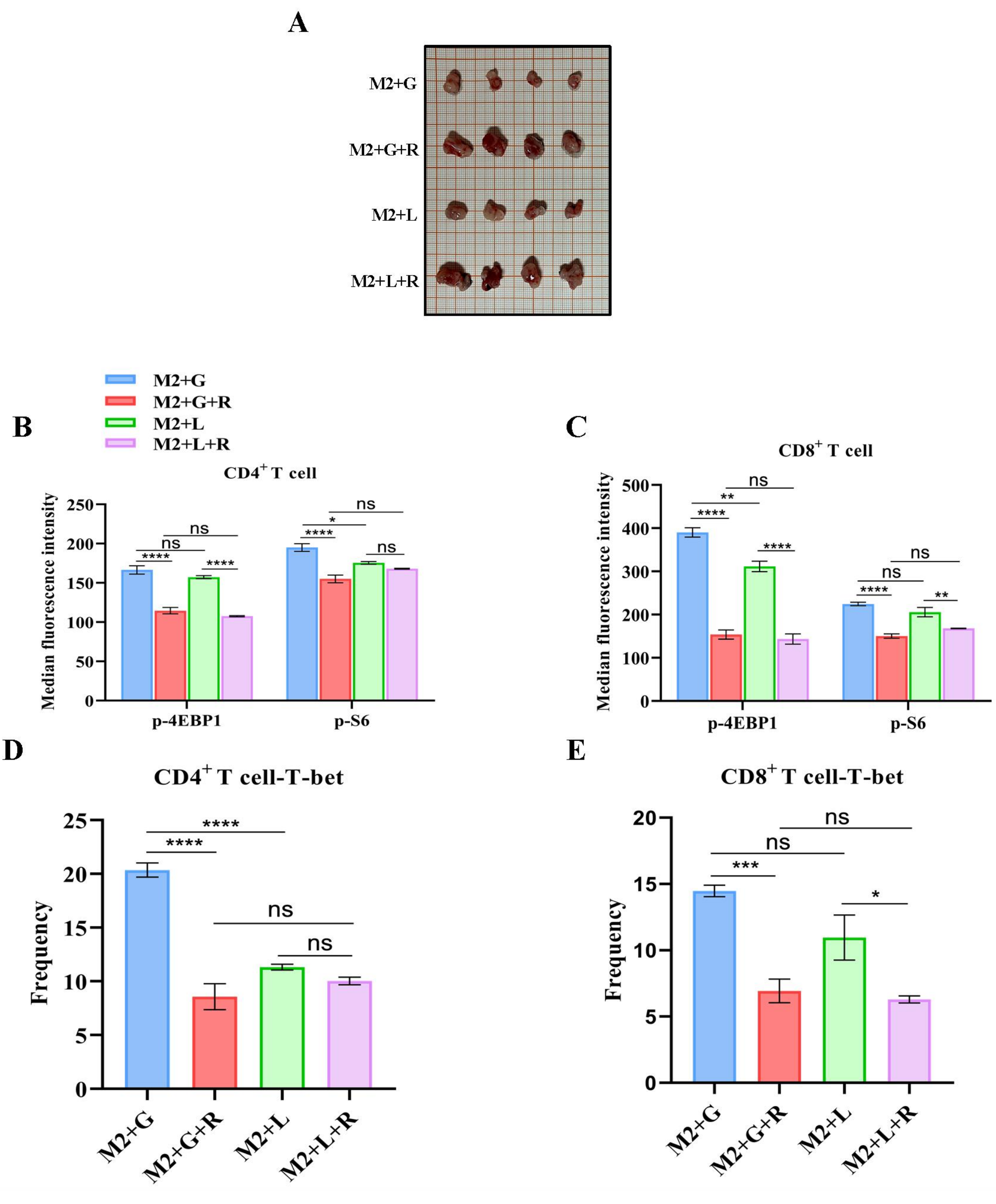

Figure. S8. Inhibition of mTOR activity ineffectively restored the anti-tumor effect of GDNPs. **a** The tumor size of M2+G, M2+G+R, M2+L and M2+L+R four groups was photographed (n=4). **b, c** Flow cytometry was used to detect the expression of p-4EBP1 and p-S6 in T cells from four groups. **d, e** The transcription factor T- bet in T cells from four groups was detected by flow

cytometry. One-way ANOVA (b, c, d, e) was used to compare results of different experimental groups for statistically significant difference ($^{*}P < 0.05$, $^{**}P < 0.01$, $^{***}P < 0.001$, $^{****}P < 0.0001$).

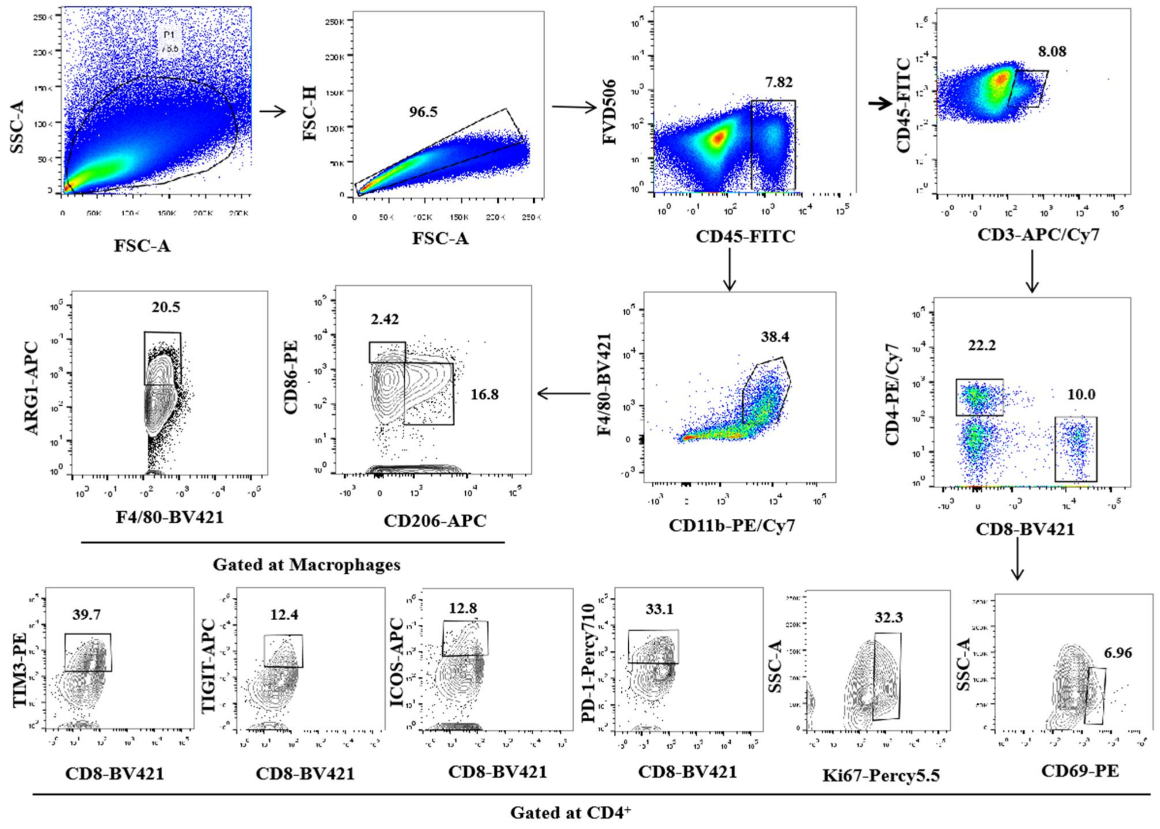

Figure. S9. Representative gating strategies for flow cytometry.

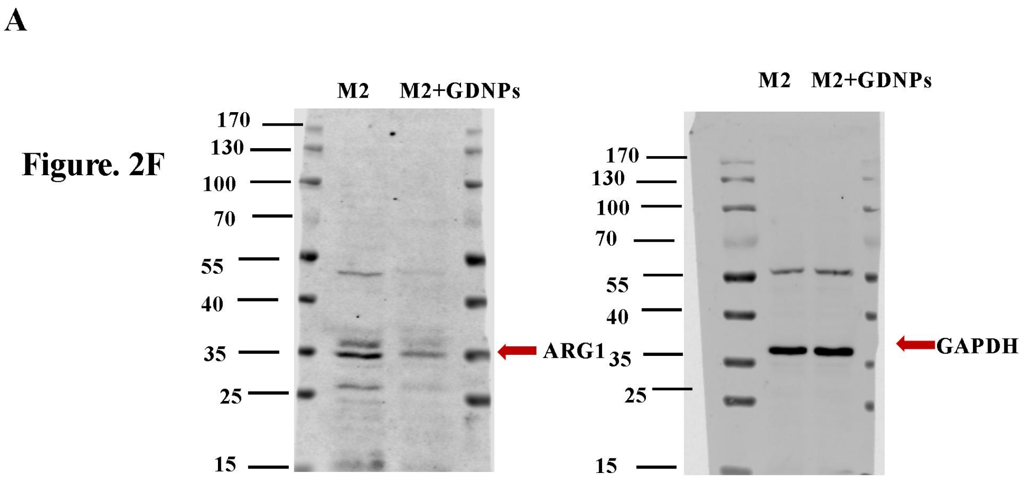

Figure. S10. Unprocessed original images of gels and western blots.
