# Supplementary material for: Ginseng-derived nanoparticles reprogram macrophages to regulate arginase-1 release for ameliorating T cell exhaustion in tumor microenvironment
Source: J Exp Clin Cancer Res. 2023 Nov 28;42:322. doi: 10.1186/s13046-023-02888-7 (PMC10683135; doi:10.1186/s13046-023-02888-7)
Supplement: Supplementary file 3 — Additional file 3: Table S1. Antibodies used for flow cytometry, western blot. Table S2. Drug information. Table S3. Experimental Models: Cell Lines. Table S4. Primer sequences for real-time RT-PCR analysis. Table S5. Effector function gene set. [file 13046_2023_2888_MOESM3_ESM.doc]

Table S1. Antibodies used for flow cytometry, western blot

| Antigen | Clone | Application | Fluorophore | Company | Catalog No. |
| --- | --- | --- | --- | --- | --- |
| CD16/32 | 93 | FC | / | Biolegend | 101302 |
| CD3 | 145-2C11 | FC | APC/Cy7 | Biolegend | 100330 |
| CD3 | 145-2C11 | FC | APC | Biolegend | 100312 |
| CD8a | 53-6.7 | FC | PE | Biolegend | 100708 |
| CD8a | QA17A07 | FC | BV421 | Biolegend | 155010 |
| CD8a | 54-6.7 | FC | APC | Biolegend | 100712 |
| CD45 | 30-F11 | FC | FITC | Biolegend | 103108 |
| CD11b | M1/70 | FC | APC/Cy7 | Biolegend | 101226 |
| F4/80 | BM8 | FC | BV421 | Biolegend | 123131 |
| CD86 | PO3 | FC | PE | Biolegend | 105106 |
| CD206 | C068C2 | FC | APC | Biolegend | 141708 |
| CD4 | GK1.5 | FC | PE/Cy7 | Biolegend | 100422 |
| CD4 | GK1.5 | FC | APC | Biolegend | 100412 |
| CD4 | GK1.5 | FC | BV421 | Biolegend | 100437 |
| Fixable Viability Dye | N/A | FC | eFluor 506 | Invitrogen | 65-0866 |
| Ki67 | B56 | FC | Percp-Cy5.5 | BD Pharmingen | 561284 |
| CD62L | MEL-14 | FC | APC | Biolegend | 104412 |
| NK1.1 | PK136 | FC | Percp-Cy5.5 | Biolegend | 108728 |
| CD44 | IM7 | FC | PE | Biolegend | 103008 |
| TIM-3 | 5D12/TIM-3 | FC | PE | BD Pharmingen | 566346 |
| TIGIT | 1G9 | FC | BV421 | BD Horizon | 565270 |
| ICOS | 7E.17G9 | FC | PE-Cy7 | Invitrogen | 25-9942-82 |
| PD-1 | J43 | FC | Percy710 | eBioscience | 46-9985-82 |
| TOX | TXRX10 | FC | PE | eBioscience | 12-6502-80 |
| T-bet | 4B10 | FC | PE/Cy7 | Invitrogen | 25-5825-82 |
| Emoes | Dan11mag | FC | PE | Invitrogen | 12-4875-82 |
| CD69 | H1.2F3 | FC | PE | Biolegend | 104507 |
| ARG1 | A1exF5 | FC | APC | Invitrogen | 17-3697-80 |
| CD11c | N418 | FC | PE | Biolegend | 117308 |
| CFSE | / | FC | / | Invitrogen | C34554 |
| Rat lgG2a K Iso Control | eBR2a | FC | APC | eBioscience | 17-4321-81 |
| Armenian Hamster lgG Isotype control | eBio299Arm | FC | PE | eBioscience | 12-4888-81 |
| Arginase-1 | / | WB/IHC | / | CST | 93668 |
| p-4EBP1 | / | WB/FC | / | CST | 2855T |
| p-S6 | / | WB/FC | / | CST | 4858S |
| GAPDH | / | WB | / | CST | 2118S |

Table S2. Drug information

| Propduct | Clone | Company | Catalog No. |
| --- | --- | --- | --- |
| Leukocyte Activation Cocktail | N/A | BD Pharmingen | 550583 |
| CD3e Monoclonal Antibody | 145-2C11 | invitrogen | 14-0031-82 |
| CD28 Monoclonal Antibody | 37.51 | invitrogen | 16-0281-82 |
| Mouse IL-4 | N/A | Peprotech | 214-14-20μg |
| Mouse IL-13 | N/A | Peprotech | 210-13-10μg |
| M-CSF | N/A | Bioworld | BK0268-10μg |
| LPS | N/A | Sigma | L2630 |
| IFN-γ | N/A | Peprotech | 31505 |
| L-Arginine | N/A | Sigma | A5006 |
| Nω-Hydroxy-nor-L-arginine | N/A | merck | 399275 |
| rapamycin | N/A | APEXBIO | A8167 |

Table S3. Experimental Models: Cell Lines

| Cell types | Source | Catalog No. |
| --- | --- | --- |
| MC38 | the Institute of Biochemistry and Cell Biology, Academy of Science (Shanghai, China) | N/A |

Table S4. Primer sequences for real-time RT-PCR analysis

| Gene | Forward | Reverse |
| --- | --- | --- |
| GAPDH | TATGTCGTGGAGTCTACTGGT | GAGTTGTCATATTTCTCGTGG |
| Arg-1 | CAGAAGAATGGAAGAGTCAG | CAGATATGCAGGGAGTC |

Table S5. Effector function gene set

| Game | Gene Set |
| --- | --- |
| Effector | Nkg7、Cxcr6、Klrc1、Ccl5、Klrd1、Ifng、Ifitm2、Ifitm1、Klre1、Gm26917、Gzma、Klrg1 |
| M2 polarization | Mrc1, Cd163 |
